# Supplementary material for: Efficacy and safety of bronchial thermoplasty in clinical practice: a prospective, longitudinal, cohort study using evidence from the UK Severe Asthma Registry
Source: BMJ Open. 2019 Jun 19;9(6):e026742. doi: 10.1136/bmjopen-2018-026742 (PMC6589003; doi:10.1136/bmjopen-2018-026742)
Supplement: Supplementary data [file bmjopen-2018-026742supp001.docx]

**Supplementary Material 1**

**Efficacy and safety of bronchial thermoplasty in clinical practice: a prospective, longitudinal, cohort study using evidence from the UK Severe Asthma Registry**

**Table S1:** Data fields added to the UK Severe Asthma Registry (SAR) to collect safety and efficacy outcomes for bronchial thermoplasty (BT)

| Date/time of bronchial thermoplasty |
| --- |
| Procedure Number |
| Inspection of previously treated sites satisfactory (N/Y) |
| Details of previously treated airways |
| Bronchial thermoplasty procedure performed (N/Y) |
| Bronchoscopist |
| Catheter Operator |
| Technician/Nurse |
| Make/model of BT device |
| Serial number of catheter |
| Any hospital admissions since last treatment (including visits to asthma clinic) (N/Y)   - (If Y): Details of asthma/BT procedure related admissions - (If Y): Details of other admissions |
| Prednisolone given for 5 days peri-procedure (N/30mg/40mg/50mg/60mg) |
| Was additional asthma medication prescribed because of this procedure (N/Y)   - (If Y): Details of other pre-treatment asthma medications |
| Site treated (RLL/LLL/RUL+LUL) |
| Number of activations |
| Missed segments: RLL (None, Posterior basal segment, Lateral basal segment, Anterior basal segment, Medial basal segment, Right lower lobe bronchus, Apical lower lobe segment) |
| Missed segments: LLL (None, Posterior basal segment, Lateral basal segment, Anterior basal segment, Left lower lobe bronchus, Apical lower lobe segment) |
| Missed segments: RUL (None, Posterior segment, Apical segment, Anterior segment, Right upper lobe bronchus) |
| Missed segments: LUL (None, Apicoposterior segment, Anterior segment, Superior division bronchus, Lingula segment, Left upper lobe bronchus) |
| Number of missed segments |
| Lowest sats peri procedure (%) |
| Duration of procedure (induction to scope out) (mins) |
| Patient discharged post procedure (N/Y)   - (If N): Duration of admission (<4hours/4-12hours/12-24hours/>24hours) - (If >24 hours): Number of days |
| Unanticipated procedural morbidity |

**Table S2:** Missing data for fields used in multiple imputation analysis

| **Field** | **BTBL missing data (n=60)** | **FU12 missing data (n=60)** |
| --- | --- | --- |
| Hospital | 0 (0%) | 0 (0%) |
| Gender | 0 (0%) | 0 (0%) |
| Age | 0 (0%) | 0 (0%) |
| BMI | 2 (3.3%) | 15 (25%) |
| Smoking status | 4 (6.7%) | 9 (15%) |
| Eosinophil count (blood) | 17 (28.3%) | 37 (61.7%) |
| FEV1 % predicted | 3 (5%) | 8 (13.3%) |
| AQLQ score | 20 (33.3%) | 23 (38.3%) |
| EQ-5D score | 31 (51.7%) | 31 (51.7%) |
| ACQ score | 23 (38.3%) | 20 (33.3%) |
| HADS score (Anxiety) | 27 (45%) | 32 (53.3%) |
| HADS score (Depression) | 27 (45%) | 32 (53.3%) |
| Rescue steroid courses | 7 (11.7%) | 5 (8.3%) |
| Unscheduled healthcare visits**^†^** | 9 (15%) | 8 (13.3%) |
| Hospital admissions | 7 (11.7%) | 5 (8.3%) |

**^†^** Includes visits to A&E, GP and asthma clinic

Original sources describing the application of the multiple imputation technique suggested that 5 to 10 imputations were sufficient [24,25]. However, a greater number has since been recommended to avoid producing a large Monte Carlo error. White et al [26] suggested a number at least as large as the percentage of missing data and Graham, Olchowski and Gilreath [27] recommend up to 100 imputations for 70% missing data. We therefore studied the levels of missing data and set the number of imputations used in the model to 100.

Default methods of imputation were used for each variable: predictive mean matching for numeric data, logistic regression for binary data, polytomous regression for unordered categorical data and proportional odds for ordered categorical data.

**Table S3:** Number of records in UKSAR at 31/10/2016

| **Time point** | **Description** | **Patient records in UKSAR** | **Patients who reached time point at 31/10/2016**  **(total/in efficacy study)** |
| --- | --- | --- | --- |
| BL | Baseline (pre BT) | 126 | - |
| BT1 | First BT procedure | 128 | - |
| BT2 | Second BT procedure | 125 | - |
| BT3 | Third BT procedure | 117 | - |
| FU6 | Follow-up visit (6 months) | 76 | 121/86 |
| FU12 | Follow-up visit (12 months) | 60 | 106/82 |
| FU24 | Follow-up visit (24 months) | 34 | 81/64 |
| FU36 | Follow-up visit (36 months) | 15 | 42/38 |
| FU48 | Follow-up visit (48 months) | 8 | 22/22 |
| FU60 | Follow-up visit (60 months) | 2 | 5/5 |

**Table S4:** Comparison of baseline characteristics of BT patients (efficacy study). Data shown as mean (sd) or median [min,LQ,UQ,max]

| **Characteristic** | **All patients with BTBL**  **(n=126)** |  | **Patients included in efficacy study (n=86)** | **Patients excluded from efficacy study**  **(n=40)** | **P value** |
| --- | --- | --- | --- | --- | --- |
| **Age at first BT (years)** | 43.7 (12.2)  n=125 |  | 41.9 (11.7)  n=86 | 47.5 (12.54)  n=39 | 0.020 |
| **Female (%)** | 69.8  n=126 |  | 68.6  (n=86) | 72.5  n=40 | 0.657 |
| **BMI (kg/m^2^)** | 31.8 (7.5)  n=119 |  | 31.2 (7.4)  n=81 | 32.9 (7.8)  n=38 | 0.256 |
| **Non-smoker/**  **Ex-smoker (%)** | 98.3  n=117 |  | 97.5  n=79 | 100  n=38 | 1.0 |
| **FEV_1_ % predicted** | 71.0 (21.8)  n=118 |  | 69.7 (21.7)  n=82 | 74.1 (22.0)  n=36 | 0.316 |
| **Eosinophil count (blood)** | 0.28 (0.34)  n=89 |  | 0.30 (0.37)  (n=63) | 0.21 (0.21)  (n=26) | 0.122 |
| **AQLQ score** | 3.66 (1.35)  n= 81 |  | 3.64 (1.26)  n= 59 | 3.71 (1.60)  n=22 | 0.848 |
| **EQ-5D score** | 0.57 (0.36)  n=54 |  | 0.53 (0.38)  n=42 | 0.72 (0.23)  n=12 | 0.049 |
| **ACQ score** | 3.19 (1.29)  n=67 |  | 3.28 (1.36)  n=49 | 2.93 (1.06)  n=18 | 0.278 |
| **HADS score (Anxiety)** | 8.13 (5.46)  n=63 |  | 8.52 (5.54)  n=48 | 6.87 (5.19)  n=15 | 0.30 |
| **HADS score (Depression)** | 6.35 (5.19)  n=63 |  | 6.46 (5.25)  n=48 | 6.0 (5.15)  n=15 | 0.767 |
| **Rescue steroid courses**  **(previous year)** | 4 [0,2,6,15]  n=100 |  | 4 [0,2,5.5,15]  n=75 | 5 [0,3,8,12]  n=25 | 0.203 |
| **Unscheduled healthcare^†^**  **(previous year)** | 5 [0,2,6,20]  n=100 |  | 5 [0,2,6,20]  n=71 | 5 [0,3,6,15]  n=29 | 0.472 |
| **Hospital admissions**  **(previous year)** | 1 [0,0,3,11]  n=103 |  | 2 [0,0,3,11]  n=76 | 1 [0,0,3,11]  n=27 | 0.490 |

**^†^** Includes visits to A&E, GP and asthma clinic

**Table S5:** Available efficacy data and summary statistics at baseline and follow-up visits. Data shown as mean (sd) or median [min,LQ,UQ,max]

|  | **BTBL (n=86)** | **FU6 (n=76)** | **FU12 (n=60)** | **FU24 (n=34)** | **FU36 (n=15)** | **FU48 (n=8)** | **FU60 (n=2)** |
| --- | --- | --- | --- | --- | --- | --- | --- |
| **AQLQ score** | 3.64 (1.26)  n=59 | 4.16 (1.47)  n=41 | 4.24 (1.45)  n=37 | 4.40 (1.62)  n=19 | 4.56 (1.57)  n=5 | 4.92 (2.11)  n=4 | -  n=0 |
| **EQ-5D score** | 0.53 (0.38)  n=42 | 0.63 (0.33)  n=30 | 0.62 (0.38)  n=29 | 0.65 (0.35)  n=18 | 0.79 (0.07)  n=4 | -  n=0 | -  n=0 |
| **ACQ score** | 3.28 (1.36)  n=49 | 2.72 (1.40)  n=47 | 2.75 (1.34)  n=40 | 3.06 (1.27)  n=21 | 2.85 (1.37)  n=6 | 3.1 (2.25)  n=3 | 5.5  n=1 |
| **HADS score (Anxiety)** | 8.52 (5.54)  n=48 | 7.73 (5.0)  n=33 | 6.46 (5.20)  n=28 | 5.28 (5.65)  n=18 | 5.80 (3.70)  n=5 | 7.0 (7.0)  n=3 | 19.0  n=1 |
| **HADS score (Depression)** | 6.46 (5.25)  n=48 | 5.94 (4.87)  n=33 | 5.07 (4.50)  n=28 | 4.67 (4.85)  n=18 | 7.0 (3.74)  n=5 | 6.33 (7.09)  n=3 | 11.0  n=1 |
| **FEV1 % predicted** | 69.65 (21.71) n=82 | 71.47 (21.62)  n=59 | 74.90 (21.34)  n=52 | 72.71 (21.08)  n=31 | 76.50 (18.78)  n=12 | 77.86 (25.39)  n=7 | 81.5 (13.44)  n=2 |
|  |  |  |  |  |  |  |  |
| **Days last BT procedure to follow-up** | - | 190  [113,172.8,210.3,285]  n=76 | 381.5  [266,363.8,408,531]  n=60 | 738.5  [571,720.3,775.8,924]  n=34 | 1118  [1059,1079.5,1160.5,1228]  n=15 | 1508.5  [1367,1479.8,1524.8,1545]  n=8 | 1821  [1787,1804,1838,1855]  n=2 |
| **Rescue steroid courses**  **(annualised)** | 4  [0,2,5.5,15]  n=75 | 2.0  [0,0,3.6,11.7]  n=67 | 3.1  [0,1.4,5.8,18.8]  n=55 | 1.9  [0,0.8,4.9,12.1]  n=29 | 3.1  [0,1.8,4.5,7.5]  n=14 | 2.9  [0,1.4,7.1,8.7]  n=8 | 5.8  [2.2,4.0,7.5,9.3]  n=2 |
| **Unscheduled healthcare^†^**  **(annualised)** | 5  [0,2,6,20]  n=71 | 2.0  [0,0,4.9,12.1]  n=61 | 3.2  [0,1.1,5.6,11.5]  n=52 | 1.3  [0,0,2.9,11.8]  n=29 | 3.0  [0,2.0,5.1,10.6]  n=14 | 4.1  [0,0,5.3,6.1]  n=8 | 6.3  [3.3,4.8,7.8,9.3]  n=2 |
| **Hospital admissions**  **(annualised)** | 2  [0,0,3,11]  n=76 | 0  [0,0,0,7.2]  n=68 | 0  [0,0,1.9,11.2]  n=55 | 0  [0,0,0.6,3.9]  n=29 | 0  [0,0,1.0,4.2]  n=14 | 0  [0,0,0,4.8]  n=8 | 0.7  [0,0.3,1.0,1.3]  n=2 |

**^†^** Includes visits to A&E, GP and asthma clinic

**Fig S1:** Efficacy data (continuous variables) at baseline and follow-ups, showing mean and 95% confidence intervals of the mean

**
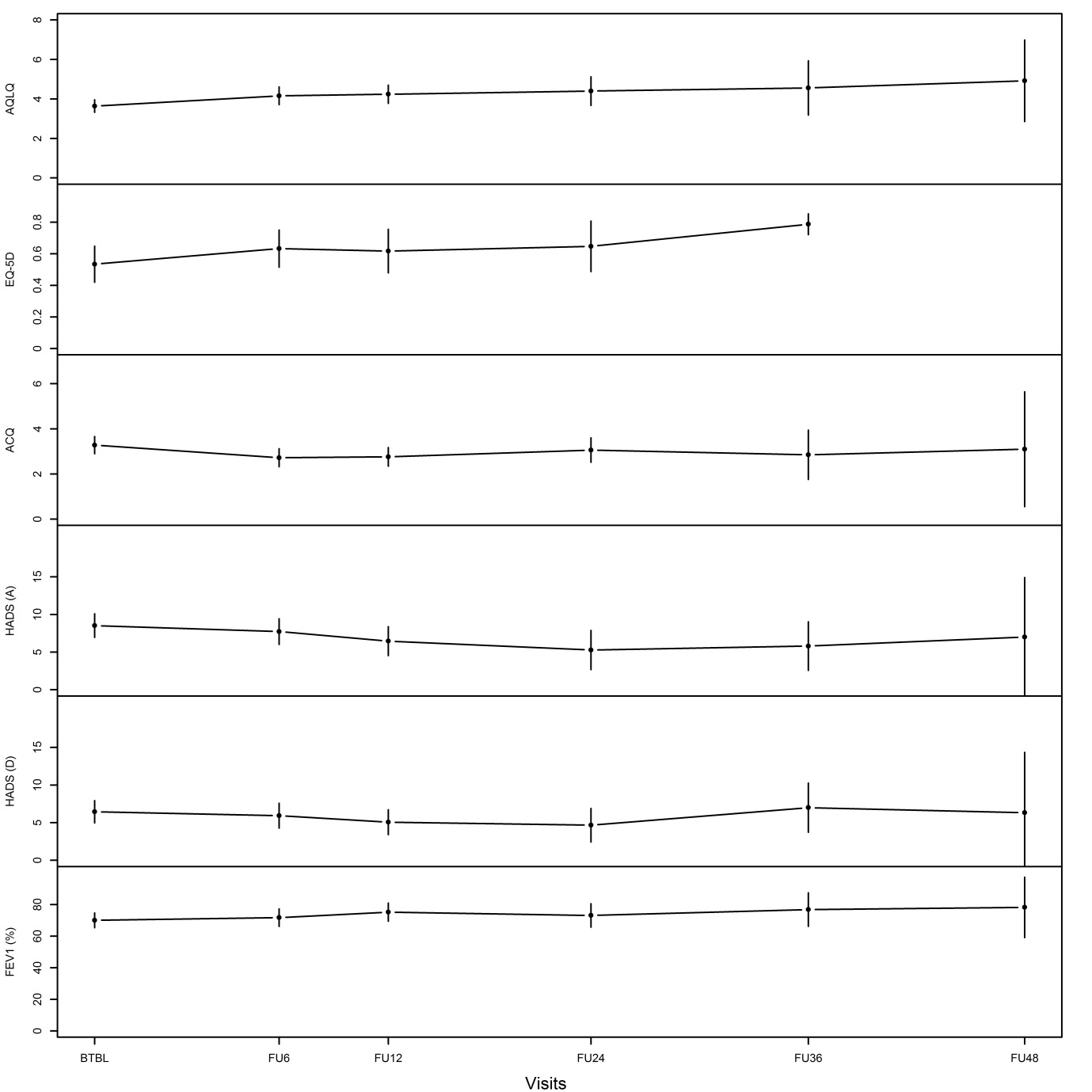
**
