# Supplementary material for: Efficacy and safety of bronchial thermoplasty in clinical practice: a prospective, longitudinal, cohort study using evidence from the UK Severe Asthma Registry
Source: BMJ Open. 2019 Jun 19;9(6):e026742. doi: 10.1136/bmjopen-2018-026742 (PMC6589003; doi:10.1136/bmjopen-2018-026742)
Supplement: Supplementary data [file bmjopen-2018-026742supp002.pdf]

Multiple Imputation using MICE package: BTBL -> FU12 AQLQ analysis:
==
Number of imputations= 100 , Number of iterations=5 (default), imputation method=default, seed=500

Columns included in imputation:

Hospital, Gender, BT1_Age, BL_BMI, BL_SmokingStatus, BL_EosinophilCount_blood, BL_FEV1_Predicted, BL_AQLQ, BL_Eq_5d, BL_ACQ, BL_HadsA, BL_HadsD, BL_RescueSteroidCourses, BL_UnscheduledHealthcareVisits, BL_TotalNumberOfHospitalAdmissions, FU12_Age, FU12_BMI, FU12_SmokingStatus, FU12_EosinophilCount_blood, FU12_FEV1_Predicted, FU12_AQLQ, FU12_Eq_5d, FU12_ACQ, FU12_HadsA, FU12_HadsD, FU12_Annualised_RescueSteroids, FU12_Annualised_UnscheduledHC, FU12_Annualised_HospAdmissions

1. Paired t-tests
=================
60 patients have a 12 month follow-up record and are included in the non-imputed/imputed analysis below

a) Paired t-test performed using non-imputed data:
==
BTBL n= 40 (mean AQLQ = 3.557)
FU12 n= 37 (mean AQLQ = 4.243)
Paired n= 28 (mean AQLQ diff= 0.7507143 , p= 0.0002593749)

b) Paired t-test performed using MICE:
======================================

	AQLQ_BTBL_mean	AQLQ_FU12_mean	AQLQ_change_mean	AQLQ_change_sd	p_value
1	3.679	4.201	0.522	1.021	2.046333e-04
2	3.626	4.234	0.608	1.398	1.320145e-03
3	3.696	4.367	0.671	0.957	1.092763e-06
4	3.418	4.089	0.671	1.001	2.744490e-06
5	3.551	4.224	0.674	1.027	4.068589e-06
6	3.508	4.239	0.731	1.014	6.228038e-07
7	3.584	4.153	0.570	1.037	7.558415e-05
8	3.526	4.143	0.617	1.160	1.194300e-04
9	3.578	4.289	0.710	1.144	1.082341e-05
10	3.529	4.255	0.726	1.027	9.422217e-07
11	3.505	4.272	0.767	0.944	4.193465e-08
12	3.623	4.038	0.415	1.353	2.091213e-02
13	3.568	4.153	0.585	1.024	4.194729e-05
14	3.525	4.075	0.550	1.141	4.224508e-04
15	3.671	4.241	0.570	1.360	1.924222e-03
16	3.559	4.150	0.592	1.114	1.225935e-04
17	3.567	4.239	0.673	1.064	7.975311e-06
18	3.555	4.410	0.855	1.041	3.212725e-08
19	3.556	4.418	0.862	1.044	2.819920e-08
20	3.645	4.318	0.673	1.116	1.780862e-05
21	3.591	4.200	0.609	0.978	1.028893e-05
22	3.677	4.202	0.525	1.028	2.058468e-04
23	3.403	4.220	0.817	1.008	4.452123e-08
24	3.499	4.087	0.588	1.096	1.061262e-04
25	3.513	4.242	0.729	1.130	5.514240e-06

26	3.368	4.361	0.992	1.228	4.745462e-08
27	3.578	4.219	0.641	0.984	4.659109e-06
28	3.554	4.156	0.602	1.174	1.955385e-04
29	3.544	4.327	0.783	1.164	2.523290e-06
30	3.549	4.268	0.718	1.135	7.738990e-06
31	3.596	4.145	0.549	1.093	2.564226e-04
32	3.579	4.296	0.717	1.104	4.923972e-06
33	3.572	4.193	0.621	0.952	4.548948e-06
34	3.583	4.336	0.753	1.037	5.419597e-07
35	3.499	4.308	0.809	1.324	1.418903e-05
36	3.883	3.937	0.054	1.660	8.031300e-01
37	3.636	4.356	0.720	1.123	6.188136e-06
38	3.627	4.189	0.562	1.243	8.864559e-04
39	3.523	4.284	0.761	1.117	1.974242e-06
40	3.711	4.327	0.616	1.100	5.726456e-05
41	3.442	4.178	0.736	1.162	7.744180e-06
42	3.571	4.053	0.482	1.093	1.168465e-03
43	3.693	4.265	0.572	1.033	6.652695e-05
44	3.614	4.287	0.673	1.135	2.321598e-05
45	3.431	4.153	0.722	1.064	2.157225e-06
46	3.764	4.263	0.498	1.098	8.500630e-04
47	3.623	4.162	0.539	1.094	3.311606e-04
48	3.469	4.204	0.734	1.047	1.099687e-06
49	3.601	4.002	0.401	1.336	2.366637e-02
50	3.652	4.245	0.593	1.185	2.698488e-04
51	3.608	4.204	0.596	1.183	2.494405e-04
52	3.846	4.321	0.476	1.323	7.174397e-03
53	3.572	4.117	0.545	1.110	3.446290e-04
54	3.533	4.511	0.978	0.981	1.574084e-10
55	3.637	4.181	0.544	1.363	3.052196e-03
56	3.470	4.147	0.677	1.056	6.109291e-06
57	3.631	4.206	0.575	1.088	1.300066e-04
58	3.626	4.214	0.587	1.163	2.387147e-04
59	3.592	4.359	0.766	1.255	1.441879e-05
60	3.459	4.306	0.847	1.086	1.111670e-07
61	3.586	4.636	1.050	1.384	2.070639e-07
62	3.647	4.437	0.790	1.176	2.597081e-06
63	3.634	4.139	0.505	1.157	1.291805e-03
64	3.444	4.135	0.691	1.349	2.005913e-04
65	3.533	4.310	0.777	1.045	3.184540e-07
66	3.564	4.285	0.721	1.042	1.425469e-06
67	3.667	4.283	0.616	0.984	9.377156e-06
68	3.415	4.263	0.848	1.114	1.880069e-07
69	3.535	4.055	0.520	1.070	3.867826e-04
70	3.567	4.230	0.662	1.087	1.508066e-05
71	3.563	4.238	0.675	1.060	7.027794e-06
72	3.629	4.324	0.694	1.075	5.403097e-06
73	3.654	4.206	0.553	1.156	4.728816e-04
74	3.529	4.322	0.793	1.124	9.885601e-07
75	3.559	4.145	0.586	1.068	7.792340e-05
76	3.597	4.182	0.586	1.250	5.977232e-04

```
77           3.627          4.365            0.738          1.083 1.969082e-06
78           3.518          4.126            0.608          0.963 8.083937e-06
79           3.521          4.233            0.712          1.208 2.599152e-05
80           3.610          4.279            0.670          0.923 5.504651e-07
81           3.534          4.138            0.604          1.080 5.789996e-05
82           3.599          4.161            0.562          1.150 3.621040e-04
83           3.619          4.208            0.589          1.457 2.695669e-03
84           3.553          4.270            0.717          1.248 3.889592e-05
85           3.647          4.381            0.734          1.346 8.421207e-05
86           3.525          4.242            0.718          0.994 6.041832e-07
87           3.590          4.230            0.640          0.943 2.132386e-06
88           3.505          4.099            0.594          1.061 5.770850e-05
89           3.469          4.248            0.779          1.038 2.615707e-07
90           3.619          4.077            0.458          1.260 6.644815e-03
91           3.571          4.133            0.561          1.146 3.526227e-04
92           3.704          4.201            0.497          0.922 9.906683e-05
93           3.722          4.488            0.766          1.278 1.969742e-05
94           3.576          4.155            0.579          1.188 3.696589e-04
95           3.577          4.250            0.674          1.322 2.128204e-04
96           3.489          4.272            0.783          1.122 1.218046e-06
97           3.590          4.259            0.670          1.001 2.797319e-06
98           3.529          4.385            0.856          0.906 7.912775e-10
99           3.664          4.403            0.738          1.124 3.974174e-06
100          3.679          4.304            0.626          1.048 2.117619e-05


Pooled results:
---------------
 AQLQ_BTBL_mean AQLQ_BTBL_sd AQLQ_FU12_mean AQLQ_FU12_sd AQLQ_change_mean AQLQ_change_sd    min_P_val max_P_val count_p_nonsig
          3.579        0.085          4.236         0.11            0.657          0.134 1.574084e-10   0.80313              1
------------------------------------------------------------------------------------------------------------------------------

2. Multiple linear regression (MLR)
===================================
60 patients have a 12 month follow-up record and are included in the non-imputed/imputed analysis below

a) MLR performed using non-imputed data (using median values for centering):
============================================================================
BTBL n= 40 (mean AQLQ = 3.557 )
FU12 n= 37 (mean AQLQ = 4.243 )
paired n = 28
MLR modelled the outcome as the change in AQLQ from BT baseline to 12 month follow-up with covariates:
age at first BT procedure, BMI at BT baseline, FEV1 (% predicted) at BT baseline and eosinophil count (blood) at BT baseline

Call:
lm(formula = AQLQdiff ~ Adj_BT1_CALC_Age + Adj_BL_CALC_Bmi + 
    Adj_BL_CALC_FEV1_Predicted + Adj_BL_EosinophilCount_blood, 
    data = AQLQ_DIFF)

Residuals:
     Min       1Q   Median       3Q      Max 
```

```
-1.31511 -0.31958 -0.02134  0.42481  1.09010

Coefficients:
                             Estimate Std. Error t value Pr(>|t|)
(Intercept)                  0.787139   0.161073   4.887 0.000165 ***
Adj_BT1_CALC_Age            -0.063336   0.012883  -4.916 0.000155 ***
Adj_BL_CALC_Bmi             -0.049952   0.025250  -1.978 0.065374 .
Adj_BL_CALC_FEV1_Predicted   0.013192   0.007707   1.712 0.106266
Adj_BL_EosinophilCount_blood -0.038687   0.526264  -0.074 0.942309
---
Signif. codes:  0 ‘***’ 0.001 ‘**’ 0.01 ‘*’ 0.05 ‘.’ 0.1 ‘ ’ 1

Residual standard error: 0.6251 on 16 degrees of freedom
  (7 observations deleted due to missingness)
Multiple R-squared:  0.6721,    Adjusted R-squared:  0.5901
F-statistic: 8.199 on 4 and 16 DF,  p-value: 0.0008522


b) MLR using MICE:
==================
                                  est          se           t       df   Pr(>|t|)       lo 95       hi 95 nmis       fmi    lambda
(Intercept)               2.255356211 1.310516486  1.72096745 27.49264 0.09649391 -0.43134875 4.942061173   NA 0.4872430    0.4512508
BT1_CALC_Age             -0.028672937 0.015566560 -1.84195714 28.99557 0.07573107 -0.06051034 0.003164465    0 0.4597051    0.4236801
BL_CALC_BMI              -0.030466048 0.024170045 -1.26048783 29.27763 0.21744708 -0.07987899 0.018946892    2 0.4545732    0.4185448
BL_CALC_FEV1_Predicted    0.008620545 0.009267073  0.93023383 27.04739 0.36047888 -0.01039236 0.027633448    3 0.4954656    0.4594883
BL_EosinophilCount_blood -0.076979354 0.788169309 -0.09766855 26.05245 0.92294303 -1.69692585 1.542967139   17 0.5139486    0.4780146
```
