# Supplementary material for: Efficacy and safety of bronchial thermoplasty in clinical practice: a prospective, longitudinal, cohort study using evidence from the UK Severe Asthma Registry
Source: BMJ Open. 2019 Jun 19;9(6):e026742. doi: 10.1136/bmjopen-2018-026742 (PMC6589003; doi:10.1136/bmjopen-2018-026742)
Supplement: Author's manuscript [file bmjopen-2018-026742.draft_revisions.pdf]

BMJ Open

BMJ Open is committed to open peer review. As part of this commitment we make the peer review history of every article we publish publicly available.

When an article is published we post the peer reviewers' comments and the authors' responses online. We also post the versions of the paper that were used during peer review. These are the versions that the peer review comments apply to.

The versions of the paper that follow are the versions that were submitted during the peer review process. They are not the versions of record or the final published versions. They should not be cited or distributed as the published version of this manuscript.

BMJ Open is an open access journal and the full, final, typeset and author-corrected version of record of the manuscript is available on our site with no access controls, subscription charges or pay-per-view fees (http://bmjopen.bmj.com).

If you have any questions on BMJ Open's open peer review process please email info.bmjopen@bmj.com

BMJ Open

# Efficacy and safety of bronchial thermoplasty in clinical practice: evidence from the UK Severe Asthma Registry



| | |
|---|---|
| Journal: | *BMJ Open* |
| Manuscript ID | bmjopen-2018-026742 |
| Article Type: | Research |
| Date Submitted by the Author: | 21-Sep-2018 |
| Complete List of Authors: | Burn, Julie; The Newcastle upon Tyne Hospitals NHS Foundation Trust, Northern Medical Physics and Clinical Engineering<br>Sims, Andrew; The Newcastle upon Tyne Hospitals NHS Foundation Trust, Northern Medical Physics and Clinical Engineering; University of Newcastle upon Tyne, Institute of Cellular Medicine<br>Patrick, Hannah; National Institute for Health and Care Excellence, Observational Data Unit<br>Heaney, Liam; Queens University Belfast, Centre for Experimental Medicine<br>Niven, Robert; Manchester Academic Health Science Centre; Manchester University NHS Foundation Trust |
| Keywords: | Asthma < THORACIC MEDICINE, Bronchoscopy < THORACIC MEDICINE, Respiratory physiology < THORACIC MEDICINE |



SCHOLARONE™
Manuscripts

For peer review only

**TITLE**

Efficacy and safety of bronchial thermoplasty in clinical practice: evidence from the UK Severe Asthma Registry

**AUTHORS**

Burn J[1], Sims AJ[1,2], Patrick H[3], Heaney LG[4], Niven RM[5,6]

[1] The Newcastle upon Tyne Hospitals NHS Foundation Trust, UK, [2] Institute of Cellular Medicine, Faculty of Medical Sciences, University of Newcastle upon Tyne, UK, [3] National Institute for Health and Care Excellence, London, UK, [4] Centre for Experimental Medicine, Queen's University of Belfast, UK, [5] Manchester Academic Health Science Centre, Manchester University, UK, [6] Manchester University NHS Foundation Trust, UK.

**CORRESPONDING AUTHOR**

Julie Burn,
Northern Medical Physics and Clinical Engineering,
The Newcastle upon Tyne Hospitals NHS Foundation Trust,
Freeman Hospital,
Newcastle upon Tyne,
NE7 7DN,
UK
julie.burn@nuth.nhs.uk
+44 (0)191 233 6161

Andrew J Sims,
Northern Medical Physics and Clinical Engineering,
The Newcastle upon Tyne Hospitals NHS Foundation Trust,
Newcastle upon Tyne,
NE7 7DN,
UK
&
Institute of Cellular Medicine,
Faculty of Medical Sciences,
University of Newcastle upon Tyne,
Newcastle upon Tyne,
UK

Hannah Patrick,
Observational Data Unit,
National Institute for Health and Care Excellence,
London,
UK

For peer review only

Liam G Heaney,
Centre for Experimental Medicine,
Queen's University of Belfast,
Belfast,
UK

Robert M Niven,
Manchester Academic Health Science Centre,
Manchester University,
Manchester,
UK
&
The North West Lung Centre,
Manchester University NHS Foundation Trust,
Manchester,
UK

**KEYWORDS:** Asthma, Bronchoscopy, Respiratory physiology, Bronchial Thermoplasty, Observational Study, Registries

**WORD COUNT:** 3533

## ABSTRACT

*Objectives*: Use data from the UK Severe Asthma Registry (UKSAR) to assess the efficacy and safety of bronchial thermoplasty (BT) in routine UK clinical practice and to identify characteristics of 'responders'.

*Design*: Prospective multi-centre registry study.

*Setting*: All (11) UK centres performing BT.

*Participants and intervention*: Patients receiving BT in the UK between 01/06/2011-30/09/2016 who had consented to data entry into UKSAR (n=133). Efficacy data was available for 86 patients with a BT baseline and at least one follow-up record. Safety data was available for 131 patients with at least one BT procedure record.

For peer review only

*Primary and secondary outcome measures*: Efficacy: AQLQ , ACQ, EuroQol, HADS anxiety and HADS depression scores, $FEV_1$ (% predicted), rescue steroid courses, unscheduled healthcare visits (A&E/Asthma clinic/GP), hospital admissions and days lost from work/school. Safety: peri-procedural events, device problems and any other safety-related findings. Responder analysis: differences in baseline characteristics of 'responders' (≥0.5 increase in AQLQ at 12 months) and 'non-responders'.

*Results*: Following Bonferroni correction for paired comparisons, mean improvement in AQLQ at 12 months follow-up compared with BT baseline was statistically and clinically significant (0.75, n=28, p=0.0003). Median reduction in hospital admissions/year after 24 months follow-up was also significant (-1.0, n=26, p<0.0001). No deterioration in $FEV_1$ was observed. From 28 patients with AQLQ data at BTBL and 12 month follow-up, there was some evidence that lower age may predict AQLQ improvement. 18.9% (70/370) of procedures and 44.5% (57/128) of patients were affected by an adverse event; only a minority were considered serious.

*Conclusions*: Improvement in AQLQ is consistent with similar findings from clinical trials. Other efficacy outcomes demonstrated improving trends without reaching statistical significance. Missing follow-up data impacted this study but multiple imputation confirmed observed AQLQ improvement. The safety review suggested BT is being performed safely in the UK.

**ARTICLE SUMMARY**

**Strengths and limitations of this study**

- The registry captured detailed morbidity and efficacy outcomes in the largest series of severe asthma patients having bronchial thermoplasty in routine UK clinical care, allowing comparison with outcomes reported from clinical trials.
- Comprehensive coverage of UK clinical practice, with almost 100% UK coverage of BT procedures being carried out post licence, thus avoiding selection bias.

For peer review only

- Improvement in AQLQ at 12 months assessed by paired analysis was confirmed with mixed effects modelling. Results from complete case analysis were confirmed with multiple imputation to account for the effect of missing data.
- Limitations include the lack of a comparator group and changes over time including prescribed drugs, comorbidities, weight and psychological factors which may affect the measured outcomes.

For peer review only

**INTRODUCTION**

Bronchial thermoplasty (BT) is an established non-pharmacological treatment for severe asthma. Thermal energy is applied to the airway wall to reduce bronchial smooth muscle, limiting its ability to contract. It is normally delivered in three bronchoscopic procedures, approximately 3-4 weeks apart. Clinical trials,[1-3] and follow-up studies,[4-6] provided early evidence of the efficacy and safety of BT but left uncertainty over whether the trial results would translate into clinical practice.

The need for more evidence from routine clinical practice was identified by a Cochrane Review in 2014,[7], in guidelines on the treatment of severe asthma, jointly published by the European Respiratory Society and the American Thoracic Society,[8], and in the United Kingdom (UK) by the National Institute for Health and Care Excellence (NICE) in 2012. NICE reviewed BT,[9] and recommended collection of safety and efficacy outcomes through the UK Severe Asthma Registry (UKSAR),[10].

UKSAR was established in 2006 to collect data on patients with severe asthma and in response to NICE's recommendations was expanded to collect data for BT. A recent study of procedural and short-term safety of BT in UK clinical practice using both evidence from UKSAR and routine health data,[11] reported that although the rate of adverse events appeared higher than reported from clinical trials, only a minority were considered significant and that the higher rate may be explained by an observed greater severity of asthma in patients being treated in this real world setting.

Several case series have been published, reporting on BT in clinical practice,[12-17]. These and an interim analysis from a large manufacturer-sponsored study,[18] all demonstrate some improvement in quality of life, reductions in exacerbations and health care resource utilisation and in general, concur with the findings of the clinical trials that BT is safe, with none raising any serious concerns. However, Thomson and Chanez,[19] recently reported on the contributions of the evidence to date in assessing the efficacy of BT in clinical practice and conclude that uncertainty remains.

The main aim of this study is to assess the longer term efficacy for BT in UK clinical practice. Secondary aims are to use experience from clinical practice to identify the characteristics of those patients most likely to benefit from BT, and to update safety evidence reported previously. It uses data collected in UKSAR and is one of the largest 'real-world' observational studies of BT to date, with all UK centres performing BT contributing data. Its design follows the recommendations of NICE Guidance,[9], and also current international efforts to improve data collection outside of a clinical trial setting for new technologies,[20,21].

## METHODS

### Registry design

Safety and efficacy outcomes and the proposed data fields to be added to the baseline, procedural and follow-up pages in UKSAR were agreed through consultation with clinicians in the UK Severe Asthma Network and with the support of NICE (Supplement 1, Table S1). Guidance on submitting data to UKSAR was issued to all centres providing BT to achieve consistent data capture. Modifications to UKSAR to include BT therapy were reported in Burn et al,[11].

### Inclusion criteria

Eligible patients were those selected to receive BT in the UK between 01/06/2011 and 30/09/2016 with data in UKSAR. For inclusion in the efficacy study, they must have received BT treatment, had a valid BT baseline (BTBL) record and at least one follow-up record.

For peer review only

**Patient and Public Involvement**

Patients and the public were not involved in the design of this study. However, the findings are presented as part of the evidence in the update of NICE interventional guidance on Bronchial Thermoplasty,[9].

**Registry coverage**

During the data collection period regular contact was maintained with the sole manufacturer of the BT device to verify that all UK centres performing BT were entering data into UKSAR. Regular contact with participating centres was also maintained to encourage data entry and to confirm BT procedure numbers.

**Data extraction**

Ethics approval for the registry was provided by the Office for Research Ethics Committees Northern Ireland (10/NIR02/37). Patients having BT between June 2011 and September 2016 were invited to give fully informed written consent for their information to be put on UKSAR. BTBL, procedure and follow-up records in UKSAR at 31st October 2016 were extracted for analysis. In accordance with registry information governance requirements the data were provided for analysis in anonymised form.

**Data cleaning**

Prior to analysis, the follow-ups nearest in time to 6, 12, 24, 36, 48 and 60 months following the last BT procedure were allocated to these follow-up points and the median times to these were calculated. Count data for rescue steroid courses, unscheduled healthcare visits, hospital admissions and days lost from work or school were annualised to compare rates before and after BT.

**Patient baseline characteristics**

Patient baseline characteristics were obtained from UKSAR including: age at first BT procedure, gender, Body Mass Index (BMI), smoking status, eosinophil count (blood), asthma status (including pre-bronchodilator forced expiratory volume in 1 second ($FEV_1$) (% predicted) and Asthma Quality of Life Questionnaire (AQLQ) score,[22], Asthma Control Questionnaire (ACQ) score, EuroQol score,

For peer review only - http://bmjopen.bmj.com/site/about/guidelines.xhtml

HADS anxiety and depression scores, rescue steroid courses, unscheduled healthcare usage, hospital admissions and days lost from work/school in the previous year. Characteristics of age, gender, $FEV_1$ and AQLQ score were compared with patients enrolled in clinical trials,[1-3]. In addition, characteristics of those included in the efficacy study were compared with those excluded to check for exclusion bias.

**Efficacy outcomes**

The efficacy outcomes considered were AQLQ , ACQ, EuroQol, HADS anxiety and HADS depression scores, $FEV_1$ (% predicted), rescue steroid courses, unscheduled healthcare visits (A&E/Asthma clinic/GP), hospital admissions and days lost from work/school.

Outcome measures at BTBL and follow-up points were calculated using all available data. Paired comparisons of outcome measures were undertaken between BTBL and both 12 and 24 month follow-ups.

**Statistical analysis**

Mean values were used for continuous variables and median values for count data. Paired comparisons were performed using: paired t-test for continuous variables; single sample bootstrap hypothesis test for count data. Comparison of groups used two sample t-tests for continuous variables and Wilcoxon rank sum test for count data. Comparisons of proportions used proportion test and fisher test. The Bonferroni method was used to adjust for multiple comparisons. Analysis was performed using the 'R' statistical programming language with a significance level of 95%.

**Missing data and additional statistical analyses**

To assess the effect of missing data, and therefore potential bias due to using available case analysis, additional statistical analyses were performed.

(i) Multiple imputation was applied to study the change in AQLQ from BTBL to the 12 month follow-up point. The R package 'MICE',[23] was used to impute missing data values in the records of the 60 patients who had both a BTBL and 12 month follow-up record. The technique allows the analyst to select parameters for the model including the fields to be imputed, the method of imputation and

For peer review only

the number of imputations, where the latter is based on the proportion of missing data,[24]. The levels of missing data were examined and the number of imputations used in the model set to 100 (Supplement 1, Table S2).

Paired t-tests were undertaken on each of the 100 imputed datasets from which the mean and standard deviation of the 'mean change in AQLQ' were calculated. The rate of 'mean change in AQLQ' < 0.5 (not clinically significant) and the rate of non-significant p-value (< 0.05) from the 100 imputations were also reported.

(ii) A linear mixed effects model was used to measure the change in AQLQ following BT by using all available observations without imputation with the R package 'lmer',[28]. This class of model accounts for repeated observations (multiple follow-ups), tolerates missing values and estimates sizes of fixed and random effects separately. The model response was AQLQ. There was one fixed effect – time, to test whether AQLQ changed over time; and two random effects: of patients on intercept (i.e. it accounted for different baseline AQLQ between patients), and of patients on slope (i.e. it accounted for different patients having different rates of change of AQLQ after treatment). The null hypothesis of there being no change in AQLQ over time was tested using a parametric bootstrap hypothesis test with a generalised likelihood ratio test statistic. Confidence intervals were estimated using bootstrapping.

**Comparison of responders with non-responders**

Using the AIR2 trial definition of a responder (≥0.5 increase in AQLQ at 12 month follow-up compared with BTBL) the baseline characteristics of responders were compared with non-responders to assess potential characteristics of those most likely to benefit from BT.

A linear model was constructed with the change in AQLQ (12 month follow-up – BTBL) as the response variable and four covariates: BT1 age, baseline BMI, baseline $FEV_1$ (% predicted), baseline eosinophil count (blood). Patients with AQLQ observations at BTBL and 12 month follow-up were included. All covariates were centred on their median values.

For peer review only

To assess the effect of missing covariates, the model was repeated using multiple imputation with the records of 60 patients who had BTBL and 12 month follow-up records. Median centering was not possible within the multiple imputation model.

**Safety outcomes**

UKSAR records added since a previous report of safety outcomes,[11] were reviewed and peri-procedural events, device problems and other potential safety findings were tabulated.

## RESULTS

**Data and study participants**

Eighty six patients who received BT treatment, had a valid BTBL record and at least one follow-up record in UKSAR, were eligible for inclusion in the efficacy analysis (Fig. 1). Data in UKSAR for 128 patients who had at least one BT procedure were available for review of safety outcomes. The total numbers of BT records available in UKSAR are shown in Supplement 1, Table S3.

**Baseline characteristics**

126 patients had a valid BTBL record (female: 69.8%; mean age at first BT: 43.7 ± 12.2 years (n=125); BMI: 31.8 ± 7.5 kg/$m^2$ (n=119); Non-smoker/Ex -smoker:  98.3% (n=117); $FEV_1$ (% predicted): 71.0 ± 21.8 (n=118)).

The baseline characteristics of the patients included in the efficacy study (n=86) and those excluded (n=40) are shown in Supplement 1 (Table S4). The mean age of those included in the study was lower than the excluded group; however after applying a Bonferroni correction this was not significant. No other significant differences were found between the 2 groups.

A comparison of a subset of baseline characteristics with data from published clinical trials,[1-3], showed that the patients in this efficacy study were on average older, with lower $FEV_1$ (% predicted) values (except for the RISA trial) and lower AQLQ scores (Table 1).

For peer review only

**Table 1: Baseline characteristics of BT patients in UKSAR compared with clinical trials**

| | UKSAR | AIR2 trial | AIR trial | RISA trial |
|---|---|---|---|---|
| **Mean age at BT1 (years)** | 43.7 ± 12.2 (n=125)<br>Range 21-74 | 40.7 ± 11.9 (†BT, n=190)<br>40.6 ± 11.9 (††C, n=98) | 39.4 ± 11.2 (BT, n=55)<br>41.7 ± 11.4 (C, n=54) | 39.1 ± 13.0 (BT, n=15)<br>42.1 ± 12.6 (C, n=17) |
| **% female** | 70 (n=126) | 57 (BT), 61 (C) | 56 (BT), 57 (C) | 60 (BT), 41(C) |
| **Pre-bronch $FEV_1$ (% predicted)** | 71.0 ± 21.8 (n=118)<br>Range: 18-109 | 77.8 ± 15.7 (BT)<br>79.7 ± 15.1 (C) | 72.7 ± 10.4 (BT)<br>76.1 ± 9.3 (C) | 62.9 ± 12.2 (BT)<br>66.4 ± 17.8 (C) |
| **AQLQ score†††** | 3.66 ± 1.35 (n=81)<br>Range 1.0 - 6.81 | 4.30 ± 1.17 (BT)<br>4.32 ± 1.21 (C) | 5.6 ± 1.1 (BT)<br>5.7 ± 0.9 (C) | 3.96 ± 1.34 (BT)<br>4.72 ± 1.06 (C) |

† BT=subjects receiving BT treatment
†† C=control subjects
†††A higher AQLQ score represents better quality of life

**Efficacy outcomes**

Efficacy outcome measures at BTBL, 12 and 24 month follow-up points are shown in Table 2 (see Supplement 1, Table S5 for data at 6, 36, 48 and 60 months). Paired comparisons showed a clinically significant (>=0.5) improvement in AQLQ after 12 months. There were also improvements in depression scores at 12 months, reduction in health care resource utilisations and hospitalisations at 12 and 24 months. Steroid courses were reduced at 12 months and more than halved at 24 months, but these changes did not reach statistical significance. After Bonferroni correction, only improvement in AQLQ after 12 months and reduction in hospital admissions at 24 months remained statistically significant.

For peer review only

**Table 2: Efficacy: all available data in UKSAR at BTBL, 12 and 24 month follow-up; paired comparisons of BTBL data with outcomes at 12 and 24 month follow-up (data shown as mean ± sd or median [range])**

| | BTBL (n=86) | FU12 (n=60) | FU24 (n=34) | BTBL to FU12 (paired) | BTBL to FU24 (paired) |
|---|---|---|---|---|---|
| **AQLQ score** | 3.64 ± 1.26 (n=59) | 4.24 ± 1.45 (n=37) | 4.40 ± 1.62 (n=19) | 0.75 (n=28, p=0.0003)† | 0.39 (n=16, p=0.148) |
| **EQ 5D score** | 0.53 ± 0.38 (n=42) | 0.62 ± 0.38 (n=29) | 0.65 ± 0.35 (n=18) | 0.008 (n=18, p=0.909) | 0.029 (n=13, p=0.706) |
| **ACQ score** | 3.28 ± 1.36 (n=49) | 2.75 ± 1.34 (n=40) | 3.06 ± 1.27 (n=21) | -0.43 (n=36, p=0.083) | -0.26 (n=19, p=0.370) |
| **HADS score (Anxiety)** | 8.52 ± 5.54 (n=48) | 6.46 ± 5.20 (n=28) | 5.28 ± 5.65 (n=18) | -1.60 (n=20, p=0.078) | -0.93 (n=14, p=0.216) |
| **HADS score (Depression)** | 6.46 ± 5.25 (n=48) | 5.07 ± 4.50 (n=28) | 4.67 ± 4.85 (n=18) | -1.60 (n=20, p=0.047) | -0.57 (n=14, p=0.336) |
| **FEV1 (% predicted)** | 69.65 ± 21.71 (n=82) | 74.90 ± 21.34 (n=52) | 72.71 ± 21.08 (n=31) | 3.51 (n=49, p=0.152) | 2.57 (n=30, p=0.560) |
| | | | | | |
| **Days from BTBL to follow-up** | - | 381.5 [266 – 531] (n=60) | 738.5 [571 – 924] (n=34) | - | - |
| **Rescue steroid courses (annualised)** | 4 [0-15] (n=75) | 3.1 [0-18.8] (n=55) | 1.9 [0-12.1] (n=29) | -0.26 (n=49, p=0.151) | -1.42 (n=27, p=0.129) |
| **Unscheduled healthcare†† (annualised)** | 5 [0-20] (n=71) | 3.2 [0-11.5] (n=52) | 1.3 [0-11.8] (n=29) | -0.93 (n=47, p=0.018) | -1.55 (n=24, p=0.031) |
| **Hospital admissions (annualised)** | 2 [0-11] (n=76) | 0 [0-11.2] (n=55) | 0 [0-3.9] (n=29) | -2.0 (n=51, p=0.050) | -1.0 (n=26, p<0.0001)† |

† Significant with Bonferroni correction applied for 9 paired comparisons (p < 0.006) at each follow-up point
†† Includes visits to A&E, GP and asthma clinic

Table 3 shows the results of paired t-tests following multiple imputation analysis for AQLQ score at 12 months compared with BTBL. 99/100 imputations demonstrated a statistically significant mean increase in AQLQ at 12 months follow-up; 92/100 imputations demonstrated a clinically significant mean increase in AQLQ (>=0.5).

For peer review only

**Table 3: Multiple Imputation (MI): Paired comparisons of AQLQ at BTBL and 12 month follow-up (FU12) compared with non-imputed data**

| | AQLQ score (BTBL) | AQLQ score (FU12) | AQLQ change | P value |
|---|---|---|---|---|
| [†] **Non-imputed data (n=28, mean ± SEM)** | 3.52 ± 0.21 | 4.27 ± 0.27 | 0.75 ± 0.07 | 0.0003 |
| [††] **MI data (n=60)** | 3.58 ± 0.09 | 4.24 ± 0.11 | 0.66 ± 0.13[†††] | < 0.05 [††††] |

[†] Paired t-test used to compare 28 patients who had AQLQ measurements at BTBL and FU12

[††] 60 patients had BTBL and FU12 records which could be used in the multiple imputation. 100 imputations were performed, resulting in 100 values for mean AQLQ score at BTBL and 12 month follow-up, and mean change in AQLQ. The means of these values are presented in the table. Columns included in the imputation: Hospital, Gender, BT1 Age, BL BMI, BL Smoking Status, BL Eosinophil Count (blood), BL FEV1 (% predicted), BL AQLQ, BL Eq_5d, BL ACQ, BL HADS (Anxiety), BL HADS (Depression), BL Rescue Steroid Courses, BL Unscheduled Healthcare Visits, BL Hospital Admissions, FU12 Age, FU12 BMI, FU12 Smoking Status, FU12 Eosinophil Count (blood), FU12 FEV1 (% predicted), FU12 AQLQ, FU12 Eq_5d, FU12 ACQ, FU12 HADS (Anxiety), FU12 HADS (Depression), FU12 Rescue Steroids (annualised), FU12 Unscheduled Healthcare Visits (annualised), FU12 Hospital Admissions (annualised)

[†††] 92/100 imputed datasets had a mean AQLQ change >= 0.5

[††††] 99/100 imputed datasets had p < 0.05

Fig. 2 shows the results of the AQLQ repeated measures model for patients with AQLQ score at BTBL and at least one follow-up. From 128 observations of AQLQ from 42 patients (median follow-up: 572.4 days), values of the fixed effects were 3.93 (95%CI 3.55 to 4.43) for baseline AQLQ and +0.19 (95% CI 0.00 to 0.36) AQLQ/year for the mean annual change of AQLQ after treatment. The random effect of baseline AQLQ due to individual patients was 1.06 (0.75 to 1.37), the random effect of individual patients on the annual change of AQLQ was 0.15 (0.02 to 0.39) AQLQ/year, and the residual error standard deviation in the model was 0.81 (0.67 to 0.92) AQLQ. The null hypothesis, of no change in AQLQ over time, was rejected at the 5% level (p=0.027). Responders (coloured blue) are those with an >= 0.5 increase in AQLQ at 12 month follow-up compared with BTBL.

There was no significant change in mean values of $FEV_1$ (% predicted) at 12 or 24 months.

The large number of missing data points and zero entries for ‘days lost from work/school’ prevented reliable analysis. Table 4 shows all available data for days lost from work or school at BTBL (assumed to represent the preceding 12 months) and 12 month follow-up (annualised). Prior to BT, 45.2% of

For peer review only

patients (19/42) reported losing at least 1 day per year, compared with 13.5% (5/37) in the year following BT; p = 0.005 by proportion test.

**Table 4: Data in UKSAR for days lost from work/school (self-reported) at BTBL and 12 month follow-up**

| Days lost from work/school data | BTBL† | FU12†† |
|---|---|---|
| Number of patients with records | 86 | 60 |
| Number of patients with days lost reported<br>- Patients reporting zero days<br>- Patients reporting > zero days | 42<br>- 23<br>- 19 (45.2%) | 37<br>- 32<br>- 5 (13.5%) |
| Number of days lost reported<br>- Median [IQR] days lost/person (for all patients reporting days lost)<br>- Median [IQR] days lost/person (for patients reporting > zero days lost) | 406<br>- 0 [0-10]<br>- 10 [7-15] | 61<br>- 0 [0-0]<br>- 13.0 [8.9-15.1] |

† Data at BTBL is assumed to represent a 12 month period
†† Data at FU12 is annualised

**Analysis of responders versus non-responders**

18/28 patients with AQLQ data at BTBL and 12 month follow-up were classified as responders (defined by >=0.5 increase in AQLQ); 10/28 patients were classified as non-responders. The baseline characteristics of both groups are shown in Table 5.

**Table 5: Responder analysis: baseline characteristics of patients who had AQLQ data at BT baseline and 12 month follow-up (a responder is defined here as having >= 0.5 increase in AQLQ at 12 month follow-up compared with BT baseline). Data shown as mean ± sd or median [range] (n).**

| Characteristic | Responders (n=18) | Non-responders (n=10) |
|---|---|---|
| Age at first BT (years) | 35.78 ± 8.7 (18) | 50.4 ± 11.0 (10) |
| Female (%) | 61.1 (18) | 60.0 (10) |
| BMI (kg/m$^2$) | 27.7 ± 5.2 (17) | 33.23 ± 10.7 (10) |
| Non-smoker/Ex-smoker (%) | 100.0 (17) | 88.9 (9) |
| $FEV_1$ (% predicted) | 65.9 ± 22.1 (18) | 62.3 ± 19.1 (10) |
| Eosinophil count (blood) | 0.29 ± 0.25 (13) | 0.51 ± 0.39 (8) |
| AQLQ score | 3.48 ± 1.19 (18) | 3.59 ± 1.02 (10) |
| EQ-5D score | 0.54 ± 0.42 (11) | 0.58 ± 0.39 (9) |

For peer review only

| | | |
|---|---|---|
| **ACQ score** | 3.59 ± 1.25 (14) | 3.07 ± 0.71 (9) |
| **HADS score (Anxiety)** | 6.20 ± 5.53 (15) | 10.33 ± 5.29 (9) |
| **HADS score (Depression)** | 4.53 ± 5.40 (15) | 7.78 ± 5.04 (9) |
| **Rescue steroid courses (previous year)** | 5 [0 – 12] (17) | 2 [0 – 7] (10) |
| **Unscheduled healthcare† (previous year)** | 4.0 [0 – 12] (16) | 3 [0 – 10] (9) |
| **Hospital admissions (previous year)** | 1.5 [0 – 10] (16) | 0 [0 – 4] (10) |
| **Days lost from work or school (previous year)** | 0 [0 – 15] (9) | 3.5 [0 – 15] (4) |

† Includes visits to A&E, GP and asthma clinic

As reported in the pivotal AIR2 trial,[1], responders in this study also had lower mean baseline AQLQ and higher mean baseline ACQ than non-responders, however multivariate analysis showed that the only significant predictor of outcome was age at first BT procedure, whereby younger patients were more likely to have a greater improvement in AQLQ at 12 month follow-up (Supplement 2).

Multiple imputation was performed for the 60 patients having records at BTBL and 12 month follow-up. However, multivariate analysis using the pooled imputed datasets just failed to confirm age (p=0.076) as a significant predictor of improvement in AQLQ at 12 month follow-up (Supplement 2).

**Safety outcomes**

There were 370 procedure records in UKSAR, for 128 patients. Table 6 summarises peri-procedural events, device problems and potential safety-related events found following manual review of all procedure and follow-up records. Overall, 18.9% of procedures and 44.5% of patients were affected by an adverse event.

For peer review only

**Table 6: Safety**

| **Peri-procedural events** | **Number of procedures affected (n=370[†])** | **Number of patients affected (n=128[††])** |
|---|---|---|
| Events preventing treatment completion (Excessive cough, discomfort and pain/bronchospasm) | 5 | 5 |
| Infection | 8 | 8 |
| Exacerbation | 13 | 11 |
| Asthma-related symptoms<br>(Drop in FEV1, wheeze, sob, low Sao2) | 24 | 19 |
| Procedure related symptoms (Bronchospasm, dry cough, chest twinges/tightness/discomfort/pain) | 20 | 16 |
| Other (Left rib fracture) | 1 | 1 |
| Other (Metabolic acidosis) | 1 | 1 |
| Other (Inflamed airways, bleeding medial basal) | 1 | 1 |
| Other (Lung collapse, one slight) | 4 | 4 |
| Other (Procedure-related bradycardia) | 1 | 1 |
| **Additional reports** | | |
| Device-related (catheters needed replacement) | 2 | 2 |
| Prolonged stay (>=7 days) with no reason given) | 3 | 2 |
| A & E attendance with no details given | 1 | 1 |
| Airway tracheomalacia reported | 2 | 2 |
| BT3 postponed 2 months due to inflamed airways & pain | 1 | 1 |
| Three procedures not able to be performed: [†††]<br>• BT1 only<br>• BT1 and BT2 only | <br><br>-<br>- | <br><br>2<br>2 |
| **CT scan reports (up to 6 month follow-up)**<br>(From 24 CT scan reports in 21 patients) | | |
| Central bronchiectasis | - | 1 |
| Other bronchiectasis | - | 2 |
| **Total events** | 87 | - |
| **Unique procedures/patients affected** | 70 (18.9%) | 57 (44.5%) |

[†] 370 procedures in total were entered with a date in UKSAR, but some had minimal procedural information recorded
[††] Three patients were excluded as they had no procedure records entered in UKSAR
[†††] In addition, 4 patients hadn't completed all 3 procedures at 30/09/2016

Some peri-procedural adverse events, although reported in the 'Unanticipated procedural morbidity' field, could be classed as anticipated. Only a minority of procedures were affected by more serious adverse events; inflamed airways/bleeding (1), significant desaturation (1), lung collapse (one slight)

For peer review only - http://bmjopen.bmj.com/site/about/guidelines.xhtml

(4), left rib fracture (1), metabolic acidosis/lactic acidosis (1), procedure-related bradycardia (1), procedure being stopped/early termination (5).

There were two recorded device problems relating to catheters; one was changed after 2 activations when a spark was noticed, in another kinking and infolding was reported after treatment of the right upper lobe.

**DISCUSSION**

In this 'real-world' study of BT, we used clinical registry data collected during routine UK clinical practice to analyse efficacy and safety outcomes. All UK centres performing BT were included and patients in this study were not subject to any specific exclusion criteria; their suitability for BT was determined on an individual basis by a multi-disciplinary team. Compared with three clinical trials, patients selected to receive BT in clinical practice were on average older, had lower baseline $FEV_1$ (except for RISA trial) and lower AQLQ scores.

The primary outcome for the AIR2 trial was AQLQ at 12 months and it showed an improvement in AQLQ of borderline statistical and below clinical significance when compared to sham. The size of the sham placebo response in that study was unprecedented and made interpretation of the findings complex. In our study, for 28 patients who had AQLQ data at BTBL and 12 month follow-up, there was a statistically and clinically significant mean improvement in AQLQ at 12 month follow-up compared with BTBL. Several articles have questioned the reliability of the reported improvement in AQLQ in the AIR2 trial,[29-31] therefore we sought further assurance of our results by conducting multiple imputation analysis for 60 patients who had both BTBL and 12 month follow-up records. This also showed a statistically and clinically significant mean improvement in AQLQ at 12 month follow-up, consistent with the non-imputed data.

Use of repeated measures analysis for 42 patients who had AQLQ data at BTBL and at least one follow-up addresses, in part, the arbitrary choice of 12 months for conventional paired analysis of

For peer review only

change in AQLQ. This approach estimated that AQLQ increased by a mean of +0.19 /year (which was significantly greater than zero) after treatment, assuming it increased linearly. The improvement at 1 year predicted by the mean rate (0.19), taking into account all follow-up observations, was less than the observed paired improvement at 12 months (0.75). This suggests that there is still uncertainty over the characteristics of the long-term persistence effect of BT beyond 12 months.

For other efficacy outcomes, the data showed trends for improvement of similar magnitude to the clinical trials however the only other statistically significant change after Bonferroni correction was the median reduction in the annual rate of hospital admissions after 24 months. The data indicate a possible reduction in days lost from work/school but we are cautious about reporting improvements as the data includes many zero entries.

Although no significant improvement in $FEV_1$ (% predicted) was shown at any follow-up point, a small increase in absolute $FEV_1$ was seen with no subsequent decline, which is consistent with other reported studies.

An exploratory responder analysis to establish whether baseline characteristics could help target patients who might benefit most from BT compared 18 responders with 10 non-responders. This showed some interesting trends, but only younger age was a significant predictor of improvement in AQLQ. This is reported with caution as multiple imputation failed to reproduce this finding.

Peri-procedural and short-term safety outcomes were previously reported using data from UKSAR and HES,[11]. That study found that 20.4% of procedures in the cohort were affected by at least one reported event (including procedural complication, emergency respiratory readmission or respiratory A&E attendance (without subsequent hospital admission) within 30 days).

The present study sought to provide an update using the data available in UKSAR. We found that 18.9% of procedures and 44.5% of patients were affected by at least one event that could be identified from procedure or follow-up records. These rates were calculated using all 370 procedure

For peer review only - http://bmjopen.bmj.com/site/about/guidelines.xhtml

records with at least the date field completed. However, some of these had no further details of the procedure so their effect on the rates is unknown. Conversely, many peri-procedural adverse events, although reported in the ‘Unanticipated procedural morbidity’ field, could have been classed as anticipated. Including only the more serious or genuinely unanticipated events in the safety analysis would decrease the rate of adverse events. Hence full details of all events were included to enable readers to judge the number and severity of events.

A strength of this study is the comprehensive coverage reported from clinical practice. We achieved almost 100% UK coverage of BT procedures being carried out post licence as confirmed by the sole manufacturer of the BT device. Data was obtained both from centres performing lower numbers of procedures and those that had previously participated in the clinical trials, thus avoiding any selection bias.

We used more than one statistical method to analyse the change in AQLQ, and in each method used the results were consistent.

A limitation of this study is the lack of comparator group so the observed improvements may be due to placebo effect. Additionally, over time, various factors affect the measured outcomes; patients are prescribed different drugs after BT, and comorbidities, weight change, and psychological factors may all have an effect. We did not study paired data beyond 24 month follow-up as there were diminishing numbers of follow-up records available for analysis and other confounding factors may become more significant.

Despite the recommendation in NICE Guidance for data collection, the biggest limitation is the poor follow-up data completion, resulting in only a small number of patients with datasets available for follow-up analysis. However the imputation model did support the observed improvement in AQLQ score at 12 month follow-up. Improvements in several other outcome measures which were non-

For peer review only

significant after correcting for multiple testing suggest that there is still a need to capture more longer-term outcome information to fully understand the effectiveness of BT.

**CONCLUSIONS**

In summary, this paper presents outcomes from the largest UK 'real-world' study of bronchial thermoplasty to date. The mean improvement in AQLQ at 12 months compared with BTBL is consistent with similar findings from clinical trials. No deterioration in $FEV_1$ (% predicted) was observed following BT and there was a significant reduction in hospital admissions at 24 month follow-up. However, improvements were not seen in all patients and an exploration of the characteristics of ‘responders’ to BT could only identify age as a possible predictor of outcome. Current and future studies looking into factors which determine those who respond well to BT will be crucial to informing patient selection. Continued data collection would also help to understand whether the improvement in AQLQ observed for some patients is maintained over a longer period.

Of the adverse events recorded, only a minority were considered significant by the treating clinician. It appears that BT is being carried out safely in the UK, however long-term safety should continue to be monitored and the decision to select a patient for BT should continue to lie with a multi-disciplinary team who can assess their history and suitability for the procedure.

**ACKNOWLEDGEMENTS**

We are grateful for the support of staff at centres performing bronchial thermoplasty and completing data entry to the UK Severe Asthma Registry: Royal Brompton Hospital (London), Manchester University NHS Foundation Trust, Gartnavel General Hospital (Glasgow), Freeman Hospital (Newcastle upon Tyne), Heartlands Hospital (Birmingham), University Hospitals of Leicester, University Hospital Southampton, Aintree University Hospital (Liverpool), Queen Alexandra Hospital (Portsmouth), Addenbrooke’s Hospital (Cambridge), Nottingham City Hospital. We are also grateful

For peer review only

for the support of Sally Welham (British Thoracic Society) who provided invaluable advice to the steering group.

**CONTRIBUTORS**

LGH is founder and co-ordinator of the UK Severe Asthma Registry Data and provided data extracts. All authors contributed equally to the design of the study. JB and AJS were responsible for analysis of the data. All authors were responsible for interpretation of the analysis. All authors drafted, revised and approved the final version of the manuscript.

**COMPETING INTERESTS**

LGH has received grant funding from Medimmune, Novartis UK, Roche/Genentech Inc, Astra Zeneca and Glaxo Smith Kline, have taken part in Advisory Boards and given lectures at meetings supported by Glaxo Smith Kline, Respivert, Merck Sharpe & Dohme, Nycomed, Boehringer Ingelheim, Novartis and Astra Zeneca. LGH has received support funding to attend International Respiratory meetings (Astra Zeneca, Chiesi, Novartis, Boehringer Ingelheim and Glaxo Smith Kline) and has taken part in asthma clinical trials (GSK, Schering Plough, Synairgen and Roche/Genentech) for which his Institution was remunerated. LGH is Academic Lead for the Medical Research Council Stratified Medicine UK Consortium in Severe Asthma which involves Industrial Partnerships with Amgen, Johnson & Johnson, Genentech / Roche, Astra Zeneca / Medimmune, Aerocrine and Vitalograph. RN was PI on several of the thermoplasty trials, and has received honoraria for lecturing & attending advisory boards from Boston Scientific.

**FUNDING**

Pilot funding for the UK Severe Asthma Registry was provided as unrestricted research grants from Astra Zeneca, GlaxoSmithKline, Novartis and Medimmune.

For peer review only

The extension of the UK Severe Asthma Registry to include bronchial thermoplasty data collection was funded by NICE. JB and AJS are employed by The Newcastle upon Tyne Hospitals NHS Foundation Trust which hosts an External Assessment Centre funded by NICE.

**ETHICAL APPROVAL**

Ethics approval for the UK Severe Asthma Registry (UKSAR) was provided by the Office for Research Ethics Committees Northern Ireland (10/NIR02/37).

**PATIENT CONSENT**

Patients having BT between June 2011 and September 2016 were invited to give fully informed written consent for their information to be put on UKSAR.

**DATA SHARING**

The registry data used in this study were provided to The Newcastle upon Tyne Hospitals NHS Foundation Trust following approval by the Registry steering committee. In accordance with registry information governance requirements, the data were provided for analysis in anonymised form. The authors are not permitted to make the data publicly available as there is a risk that patients could be identified due to the small numbers involved.

**REFERENCES**

1. Castro M, Rubin AS, Laviolette M, Fiterman J, De Andrade Lima M, Shah PL, et al. Effectiveness and safety of bronchial thermoplasty in the treatment of severe asthma: a multicenter, randomized, double-blind, sham-controlled clinical trial. *Am J Respir Crit Care Med*. 2010;181(2):116-24.
2. Cox G, Thomson NC, Rubin AS, Niven RM, Corris PA, Siersted HC, et al. Asthma control during the year after bronchial thermoplasty. *N Engl J Med.* 2007;356:1327-37.

For peer review only

3. Pavord ID, Cox G, Thomson NC, Rubin AS, Corris PA, Niven RM, et al. Safety and efficacy of bronchial thermoplasty in symptomatic, severe asthma. *Am J Respir Crit Care Med*. 2007;176(12):1185-91.
4. Wechsler ME, Laviolette M, Rubin AS, Fiterman J, Lapa e Silva JR, Shah PL, et al. Bronchial thermoplasty: Long-term safety and effectiveness in patients with severe persistent asthma. *J Allergy Clin Immunol.* 2013;132(6):1295-302.
5. Thomson NC, Rubin AS, Niven RM, Corris PA, Siersted HC, Olivenstein R, et al. Long-term (5 year) safety of bronchial thermoplasty: Asthma Intervention Research (AIR) trial. *BMC Pulm Med*. 2011;11:8.
6. Pavord ID, Thomson NC, Niven RM, Corris PA, Chung KF, Cox G, et al. Safety of bronchial thermoplasty in patients with severe refractory asthma. *Ann Allergy Asthma Immunol*. 2013;111(5):402-7.
7. Torrego A, Solà I, Munoz AM, Roqué i Figuls M, Yepes-Nuñez JJ, Alonso-Coello P, et al. Bronchial thermoplasty for moderate or severe persistent asthma in adults. *Cochrane Database Syst Rev.* 2014; Issue 3.
8. Chung KF, Wenzel SE, Brozek JL, Bush A, Castro M, Sterk PJ, et al. International ERS/ATS guidelines on definition, evaluation and treatment of severe asthma. *Eur Respir J.* 2014;43(2):343-73.
9. IPG419 Bronchial thermoplasty for severe asthma [Internet]. NICE; [published January 2012, date accessed August 2018]. Available from https://www.nice.org.uk/guidance/ipg419
10. Heaney LG, Brightling CE, Menzies-Gow A, Stevenson M and Niven RM. Refractory asthma in the UK: cross-sectional findings from a UK multicentre registry. *Thorax* 2010;65:787-794.
11. Burn J, Sims AJ, Keltie K, Patrick H, Welham S, Heaney LG, Niven RM. Procedural and short-term safety of bronchial thermoplasty in clinical practice: evidence from a national registry and Hospital Episode Statistics. *J Asthma* 2017;54(8):872-879.

For peer review only

12. Doeing DC, Mahajan AK, White SR, Naureckas ET, Krishnan JA, Hogarth DK. Safety and feasibility of bronchial thermoplasty in asthma patients with very severe fixed airflow obstruction: a case series. *J Asthma*. 2013;50(2):215-8.

13. Fernández-Bussy S, Labarca G, Caviedes I, Folch E, Majid A. Bronchial thermoplasty for severe asthma: Initial experience in Chile. *Arch Bronconeumol*. 2016;52:52.

14. Bicknell S, Chaudhuri R, Lee N, Shepherd M, Spears M, Pitman N, et al. Effectiveness of bronchial thermoplasty in severe asthma in 'real life' patients compared with those recruited to clinical trials in the same centre. *Ther Adv Respir Dis.* 2015; 9(6):267-71.

15. Langton, David, Sha, Joy, Ing, Alvin, Fielding, David and Wood, Erica (2017). Bronchial thermoplasty in severe asthma in Australia. *Internal Medicine Journal* 19: 19.

16. Watchorn, D. C., Sahadevan, A., Egan, J. J. and Lane, S. J. (2016). The Efficacy of Bronchial Thermoplasty for Severe Persistent Asthma: The First National Experience. *Irish Medical Journal* 109(5): 406

17. Arrigo, Rita, Failla, Giuseppe, Scichilone, Nicola, La Sala, Alba, Galeone, Carla, Battaglia, Salvatore, Benfante, Alida and Facciolongo, Nicola (2016). How Effective and Safe Is Bronchial Thermoplasty in "Real Life" Asthmatics Compared to Those Enrolled in Randomized Clinical Trials? *BioMed Research International* 2016: 9132198

18. Chupp G, Laviolette M, Cohn L, McEvoy C, Bansal S, Shifren A, et al. Long-term outcomes of bronchial thermoplasty in subjects with severe asthma: a comparison of 3-year follow-up results from two prospective multicentre studies. *Eur Respir J.* 2017; 50(2).

19. Thomson NC and Chanez P. How effective is bronchial thermoplasty for severe asthma in clinical practice? *Eur Respir J.* 2017; 50: 1701140.

20. Barkun JS, Aronson JK, Feldman LS, Maddern GJ, Strasberg SM, Altman DG, et al. Evaluation and stages of surgical innovations. *Lancet* 2009; 374:1089-1096.

21. Commissioning through Evaluation [Internet]. NHS England; [date accessed August 2018]. Available from https://www.england.nhs.uk/commissioning/spec-services/npc-crg/comm-eval/

For peer review only

22. Juniper E, Guhatt G, Epstein R, Ferrie PJ, Jaeschke R, Hiller TK. Evaluation of impairment of health related quality of life in asthma: development of a questionnaire for use in clinical trials. *Thorax* 1992;47:76–83.

23. R Core Team. R: A language and environment for statistical computing [Internet]. R Foundation for Statistical Computing, Vienna, Austria; [date accessed August 2018]. Available from http://www.R-project.org/

24. Rubin DB. Multiple imputation for nonresponse in surveys. New York: John Wiley, 1987.

25. Schafer JL. Multiple imputation: a primer. *Stat Methods Med Res*. 1999 Mar;8(1):3-15.

26. White IR, Royston P, Wood AM. Multiple imputation using chained equations: Issues and guidance for practice. *Stat Med*. 2011;30(4):377–99

27. Graham, J.W., Olchowski, A.E. & Gilreath, T.D. *Prev Sci* (2007) 8: 206.

28. Pinheiro JC and Bates DM. Mixed effects models in S and S-PLUS, Springer-Verlag, New York, 2000.

29. Bel EH. Bronchial thermoplasty: has the promise been met? *Am J Respir Crit Care Med*. 2010;181(2):101-2.

30. Bel EH, Zwinderman AH. Outcome Reporting in Asthma Research. *Am J Respir Crit Care Med*. 2011;183(1): 132-3.

31. Iyer VN, Lim KG. Bronchial thermoplasty: reappraising the evidence (or lack thereof). *Chest*. 2014;146(1):17-21.

## FIGURE CAPTIONS

**Fig 1: Efficacy study participants**

**Fig 2: AQLQ series for patients with AQLQ data at BT baseline and at least one follow-up. Horizontal dashed lines show +/- 0.5 (clinically significant) change in AQLQ from BT baseline. Responders (defined as having >= 0.5 increase in AQLQ at 12 month follow-up compared with BT baseline) are shown in blue. Non-responders are shown in black.**

For peer review only

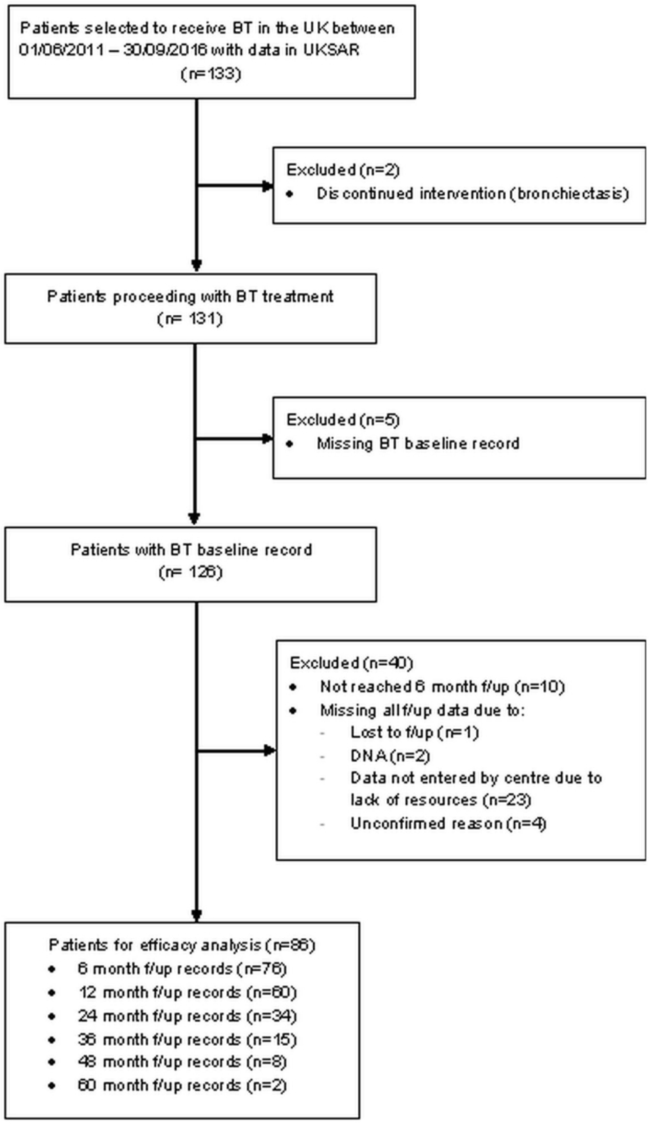


Fig 1: Efficacy study participants

130x180mm (300 x 300 DPI)

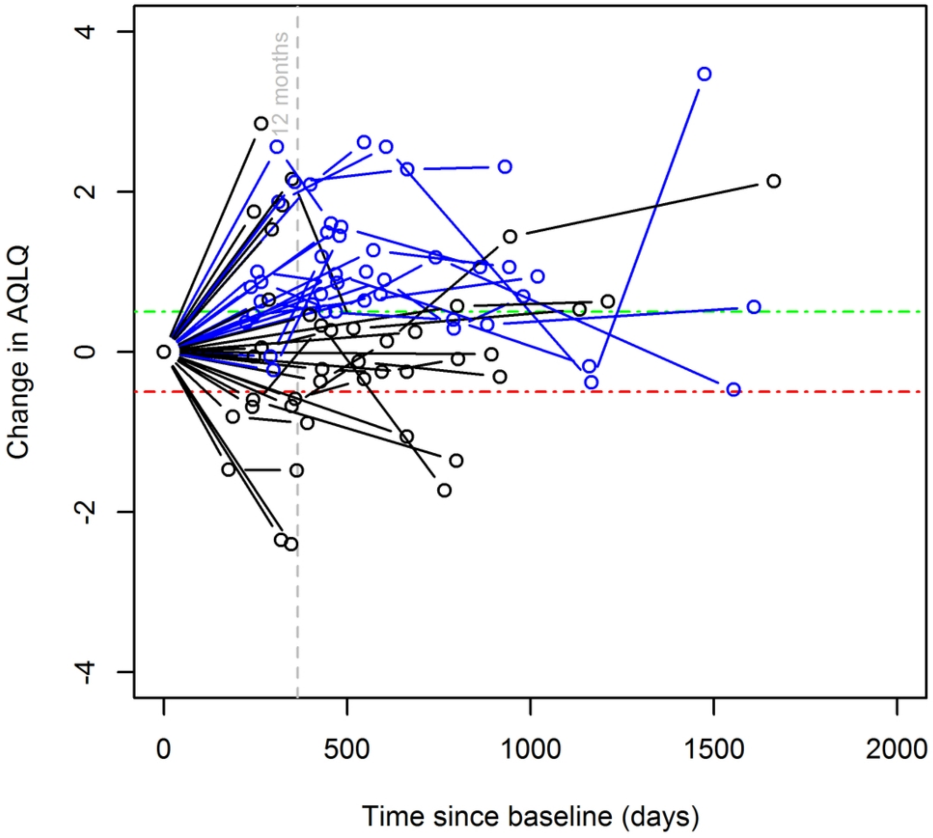


Fig 2: AQLQ series for patients with AQLQ data at BT baseline and at least one follow-up. Horizontal dashed lines show +/- 0.5 (clinically significant) change in AQLQ from BT baseline. Responders (defined as having >= 0.5 increase in AQLQ at 12 month follow-up compared with BT baseline) are shown in blue. Non-responders are shown in black.

99x99mm (300 x 300 DPI)

**Supplementary Material 1**

**Efficacy of bronchial thermoplasty in clinical practice: evidence from the UK Severe Asthma Registry (SAR)**

**Table S1: Data fields added to the UK Severe Asthma Registry (SAR) to collect safety and efficacy outcomes for bronchial thermoplasty (BT)**

| |
|---|
| Date/time of bronchial thermoplasty |
| Procedure Number |
| Inspection of previously treated sites satisfactory (N/Y) |
| Details of previously treated airways |
| Bronchial thermoplasty procedure performed (N/Y) |
| Bronchoscopist |
| Catheter Operator |
| Technician/Nurse |
| Make/model of BT device |
| Serial number of catheter |
| Any hospital admissions since last treatment (including visits to asthma clinic) (N/Y)<br>- (If Y): Details of asthma/BT procedure related admissions<br>- (If Y): Details of other admissions |
| Prednisolone given for 5 days peri-procedure (N/30mg/40mg/50mg/60mg) |
| Was additional asthma medication prescribed because of this procedure (N/Y)<br>- (If Y): Details of other pre-treatment asthma medications |
| Site treated (RLL/LLL/RUL+LUL) |
| Number of activations |
| Missed segments: RLL (None, Posterior basal segment, Lateral basal segment, Anterior basal segment, Medial basal segment, Right lower lobe bronchus, Apical lower lobe segment) |
| Missed segments: LLL (None, Posterior basal segment, Lateral basal segment, Anterior basal segment, Left lower lobe bronchus, Apical lower lobe segment) |
| Missed segments: RUL (None, Posterior segment, Apical segment, Anterior segment, Right upper lobe bronchus) |
| Missed segments: LUL (None, Apicoposterior segment, Anterior segment, Superior division bronchus, Lingula segment, Left upper lobe bronchus) |
| Number of missed segments |
| Lowest sats peri procedure (%) |
| Duration of procedure (induction to scope out) (mins) |
| Patient discharged post procedure (N/Y)<br>- (If N): Duration of admission (<4hours/4-12hours/12-24hours/>24hours)<br>- (If >24 hours): Number of days |
| Unanticipated procedural morbidity |

For peer review only

**Table S2: Missing data for fields used in multiple imputation analysis**

| Field | BTBL missing data (n=60) | FU12 missing data (n=60) |
|---|---|---|
| Hospital | 0 (0%) | 0 (0%) |
| Gender | 0 (0%) | 0 (0%) |
| Age | 0 (0%) | 0 (0%) |
| BMI | 2 (3.3%) | 15 (25%) |
| Smoking status | 4 (6.7%) | 9 (15%) |
| Eosinophil count (blood) | 17 (28.3%) | 37 (61.7%) |
| FEV1 % predicted | 3 (5%) | 8 (13.3%) |
| AQLQ score | 20 (33.3%) | 23 (38.3%) |
| EQ-5D score | 31 (51.7%) | 31 (51.7%) |
| ACQ score | 23 (38.3%) | 20 (33.3%) |
| HADS score (Anxiety) | 27 (45%) | 32 (53.3%) |
| HADS score (Depression) | 27 (45%) | 32 (53.3%) |
| Rescue steroid courses | 7 (11.7%) | 5 (8.3%) |
| Unscheduled healthcare visits[ŧ] | 9 (15%) | 8 (13.3%) |
| Hospital admissions | 7 (11.7%) | 5 (8.3%) |

[ŧ] Includes visits to A&E, GP and asthma clinic

Original sources describing the application of the multiple imputation technique suggested that 5 to 10 imputations were sufficient [24,25]. However, a greater number has since been recommended to avoid producing a large Monte Carlo error. White et al [26] suggested a number at least as large as the percentage of missing data and Graham, Olchowski and Gilreath [27] recommend up to 100 imputations for 70% missing data. We therefore studied the levels of missing data and set the number of imputations used in the model to 100.

Default methods of imputation were used for each variable:  predictive mean matching for numeric data, logistic regression for binary data, polytomous regression for unordered categorical data and proportional odds for ordered categorical data.

**Table S3: Number of records in UKSAR at 31/10/2016**

| Record type | Description | Patient records in UKSAR | Patients reached time point at 31/10/2016 |
|---|---|---|---|
| BL | Baseline (pre BT) | 126 | - |
| BT1 | First BT procedure | 128 | - |
| BT2 | Second BT procedure | 125 | - |
| BT3 | Third BT procedure | 117 | - |
| FU6 | Follow-up visit (6 months) | 76 | 86 |
| FU12 | Follow-up visit (12 months) | 60 | 82 |
| FU24 | Follow-up visit (24 months) | 34 | 64 |
| FU36 | Follow-up visit (36 months) | 15 | 38 |
| FU48 | Follow-up visit (48 months) | 8 | 22 |
| FU60 | Follow-up visit (60 months) | 2 | 5 |

For peer review only

**Table S4: Comparison of baseline characteristics of BT patients (efficacy study)**

| Characteristic | All patients with BTBL (n=126) | | Patients included in efficacy study (n=86) | Patients excluded from efficacy study (n=40) | P value |
|---|---|---|---|---|---|
| Age at first BT (years) | 43.7 ± 12.2 (n=125) | | 41.9 ± 11.7 (n=86) | 47.5 ± 12.54 (n=39) | 0.020 |
| Female (%) | 69.8 (n=126) | | 68.6 (n=86) | 72.5 (n=40) | 0.657 |
| BMI (kg/m$^2$) | 31.8 ± 7.5 (n=119) | | 31.2 ± 7.4 (n=81) | 32.9 ± 7.8 (n=38) | 0.256 |
| Non-smoker/Ex smoker (%) | 98.3 (n=117) | | 97.5 (n=79) | 100 (n=38) | 1.0 |
| FEV$_1$ % predicted | 71.0 ± 21.8 (n=118) | | 69.7 ± 21.7 (n=82) | 74.1 ± 22.0 (n=36) | 0.316 |
| Eosinophil count (blood) | 0.28 ± 0.34 (n=89) | | 0.30 ± 0.37 (n=63) | 0.21 ± 0.21 (n=26) | 0.122 |
| AQLQ score | 3.66 ± 1.35 n= (81) | | 3.64 ± 1.26 n= (59) | 3.71 ± 1.60 (n=22) | 0.848 |
| EEQ 5D score | 0.57 ± 0.36 (n=54) | | 0.53 ± 0.38 (n=42) | 0.72 ± 0.23 (n=12) | 0.049 |
| ACQ score | 3.19 ± 1.29 (n=67) | | 3.28 ± 1.36 (n=49) | 2.93 ± 1.06 (n=18) | 0.278 |
| HADS score (Anxiety) | 8.13 ± 5.46 (n=63) | | 8.52 ± 5.54 (n=48) | 6.87 ± 5.19 (n=15) | 0.30 |
| HADS score (Depression) | 6.35 ± 5.19 (n=63) | | 6.46 ± 5.25 (n=48) | 6.0 ± 5.15 (n=15) | 0.767 |
| Rescue steroid courses (previous year) | 4 [0 – 15] (n=100) | | 4 [0 – 15] (n=75) | 5 [0 – 12] (n=25) | 0.203 |
| Unscheduled healthcare[†] (previous year) | 5 [0 – 20] (n=100) | | 5 [0 – 20] (n=71) | 5 [0 – 15] (n=29) | 0.472 |
| Hospital admissions (previous year) | 1 [0 – 11] (n=103) | | 2 [0 – 11] (n=76) | 1 [0 – 11] (n=27) | 0.490 |

[†] Includes visits to A&E, GP and asthma clinic

For peer review only

**Table S5: Available efficacy data and summary statistics at baseline and follow-up visits (mean ± sd or median [range])**

| | BTBL (n=86) | FU6 (n=76) | FU12 (n=60) | FU24 (n=34) | FU36 (n=15) | FU48 (n=8) | FU60 (n=2) |
|---|---|---|---|---|---|---|---|
| **AQLQ score** | 3.64 ± 1.26 (n=59) | 4.16 ± 1.47 (n=41) | 4.24 ± 1.45 (n=37) | 4.40 ± 1.62 (n=19) | 4.56 ± 1.57 (n=5) | 4.92 ± 2.11 (n=4) | - (n=0) |
| **EQ-5D score** | 0.53 ± 0.38 (n=42) | 0.63 ± 0.33 (n=30) | 0.62 ± 0.38 (n=29) | 0.65 ± 0.35 (n=18) | 0.79 ± 0.07 (n=4) | - (n=0) | - (n=0) |
| **ACQ score** | 3.28 ± 1.36 (n=49) | 2.72 ± 1.40 (n=47) | 2.75 ± 1.34 (n=40) | 3.06 ± 1.27 (n=21) | 2.85 ± 1.37 (n=6) | 3.1 ± 2.25 (n=3) | 5.5 (n=1) |
| **HADS score (Anxiety)** | 8.52 ± 5.54 (n=48) | 7.73 ± 5.0 (n=33) | 6.46 ± 5.20 (n=28) | 5.28 ± 5.65 (n=18) | 5.80 ± 3.70 (n=5) | 7.0 ± 7.0 (n=3) | 19.0 (n=1) |
| **HADS score (Depression)** | 6.46 ± 5.25 (n=48) | 5.94 ± 4.87 (n=33) | 5.07 ± 4.50 (n=28) | 4.67 ± 4.85 (n=18) | 7.0 ± 3.74 (n=5) | 6.33 ± 7.09 (n=3) | 11.0 (n=1) |
| **FEV1 % predicted** | 69.65 ± 21.71 (n=82) | 71.47 ± 21.62 (n=59) | 74.90 ± 21.34 (n=52) | 72.71 ± 21.08 (n=31) | 76.50 ± 18.78 (n=12) | 77.86 ± 25.39 (n=7) | 81.5 ± 13.44 (n=2) |
| | | | | | | | |
| **Days BTBL to follow-up** | - | 190 [113 - 285] (n=76) | 381.5 [266 - 531] (n=60) | 738.5 [571 - 924] (n=34) | 1118 [1059 - 1228] (n=15) | 1508.5 [1367 - 1545] (n=8) | 1821 [1787 - 1855] (n=2) |
| **Rescue steroid courses (annualised)** | 4 [0-15] (n=75) | 2.0 [0-11.7] (n=67) | 3.1 [0-18.8] (n=55) | 1.9 [0-12.1] (n=29) | 3.1 [0-7.5] (n=14) | 2.9 [0-8.7] (n=8) | 5.8 [2.2-9.3] (n=2) |
| **Unscheduled healthcare† (annualised)** | 5 [0-20] (n=71) | 2.0 [0-12.1] (n=61) | 3.2 [0-11.5] (n=52) | 1.3 [0-11.8] (n=29) | 3.0 [0-10.6] (n=14) | 4.1 [0-6.1] (n=8) | 6.3 [3.3-9.3] (n=2) |
| **Hospital admissions (annualised)** | 2 [0-11] (n=76) | 0 [0-7.2] (n=68) | 0 [0-11.2] (n=55) | 0 [0-3.9] (n=29) | 0 [0-4.2] (n=14) | 0 [0-4.8] (n=8) | 0.7 [0-1.3] (n=2) |

† Includes visits to A&E, GP and asthma clinic

For peer review only

```
Multiple Imputation using MICE package: BTBL -> FU12 AQLQ analysis:
======================================================================
**Number of imputations= 100 , Number of iterations=5 (default), imputation method=default, seed=500**

Columns included in imputation:
-------------------------------
Hospital, Gender, BT1_Age, BL_BMI, BL_SmokingStatus, BL_EosinophilCount_blood, BL_FEV1_Predicted, BL_AQLQ,
BL_Eq_5d, BL_ACQ, BL_HadsA, BL_HadsD, BL_RescueSteroidCourses, BL_UnscheduledHealthcareVisits,
BL_TotalNumberOfHospitalAdmissions, FU12_Age, FU12_BMI, FU12_SmokingStatus, FU12_EosinophilCount_blood,
FU12_FEV1_Predicted, FU12_AQLQ, FU12_Eq_5d, FU12_ACQ, FU12_HadsA, FU12_HadsD, FU12_Annualised_RescueSteroids,
FU12_Annualised_UnscheduledHC, FU12_Annualised_HospAdmissions

1. Paired t-tests
=================
60 patients have a 12 month follow-up record and are included in the non-imputed/imputed analysis below

a) Paired t-test performed using non-imputed data:
==================================================
BTBL n= 40 (mean AQLQ = 3.557 )
FU12 n= 37 (mean AQLQ = 4.243 )
Paired n= 28 (mean AQLQ diff= 0.7507143 , p= 0.0002593749 )


b) Paired t-test performed using MICE:
======================================
```

| | AQLQ_BTBL_mean | AQLQ_FU12_mean | AQLQ_change_mean | AQLQ_change_sd | p_value |
|---|---|---|---|---|---|
| 1 | 3.679 | 4.201 | 0.522 | 1.021 | 2.046333e-04 |
| 2 | 3.626 | 4.234 | 0.608 | 1.398 | 1.320145e-03 |
| 3 | 3.696 | 4.367 | 0.671 | 0.957 | 1.092763e-06 |
| 4 | 3.418 | 4.089 | 0.671 | 1.001 | 2.744490e-06 |
| 5 | 3.551 | 4.224 | 0.674 | 1.027 | 4.068589e-06 |
| 6 | 3.508 | 4.239 | 0.731 | 1.014 | 6.228038e-07 |
| 7 | 3.584 | 4.153 | 0.570 | 1.037 | 7.558415e-05 |
| 8 | 3.526 | 4.143 | 0.617 | 1.160 | 1.194300e-04 |
| 9 | 3.578 | 4.289 | 0.710 | 1.144 | 1.082341e-05 |
| 10 | 3.529 | 4.255 | 0.726 | 1.027 | 9.422217e-07 |
| 11 | 3.505 | 4.272 | 0.767 | 0.944 | 4.193465e-08 |
| 12 | 3.623 | 4.038 | 0.415 | 1.353 | 2.091213e-02 |
| 13 | 3.568 | 4.153 | 0.585 | 1.024 | 4.194729e-05 |
| 14 | 3.525 | 4.075 | 0.550 | 1.141 | 4.224508e-04 |
| 15 | 3.671 | 4.241 | 0.570 | 1.360 | 1.924222e-03 |
| 16 | 3.559 | 4.150 | 0.592 | 1.114 | 1.225935e-04 |
| 17 | 3.567 | 4.239 | 0.673 | 1.064 | 7.975311e-06 |
| 18 | 3.555 | 4.410 | 0.855 | 1.041 | 3.212725e-08 |
| 19 | 3.556 | 4.418 | 0.862 | 1.044 | 2.819920e-08 |
| 20 | 3.645 | 4.318 | 0.673 | 1.116 | 1.780862e-05 |
| 21 | 3.591 | 4.200 | 0.609 | 0.978 | 1.028893e-05 |
| 22 | 3.677 | 4.202 | 0.525 | 1.028 | 2.058468e-04 |
| 23 | 3.403 | 4.220 | 0.817 | 1.008 | 4.452123e-08 |
| 24 | 3.499 | 4.087 | 0.588 | 1.096 | 1.061262e-04 |
| 25 | 3.513 | 4.242 | 0.729 | 1.130 | 5.514240e-06 |

| | | | | | |
|---|---|---|---|---|---|
| 26 | 3.368 | 4.361 | 0.992 | 1.228 | 4.745462e-08 |
| 27 | 3.578 | 4.219 | 0.641 | 0.984 | 4.659109e-06 |
| 28 | 3.554 | 4.156 | 0.602 | 1.174 | 1.955385e-04 |
| 29 | 3.544 | 4.327 | 0.783 | 1.164 | 2.523290e-06 |
| 30 | 3.549 | 4.268 | 0.718 | 1.135 | 7.738990e-06 |
| 31 | 3.596 | 4.145 | 0.549 | 1.093 | 2.564226e-04 |
| 32 | 3.579 | 4.296 | 0.717 | 1.104 | 4.923972e-06 |
| 33 | 3.572 | 4.193 | 0.621 | 0.952 | 4.548948e-06 |
| 34 | 3.583 | 4.336 | 0.753 | 1.037 | 5.419597e-07 |
| 35 | 3.499 | 4.308 | 0.809 | 1.324 | 1.418903e-05 |
| 36 | 3.883 | 3.937 | 0.054 | 1.660 | 8.031300e-01 |
| 37 | 3.636 | 4.356 | 0.720 | 1.123 | 6.188136e-06 |
| 38 | 3.627 | 4.189 | 0.562 | 1.243 | 8.864559e-04 |
| 39 | 3.523 | 4.284 | 0.761 | 1.117 | 1.974242e-06 |
| 40 | 3.711 | 4.327 | 0.616 | 1.100 | 5.726456e-05 |
| 41 | 3.442 | 4.178 | 0.736 | 1.162 | 7.744180e-06 |
| 42 | 3.571 | 4.053 | 0.482 | 1.093 | 1.168465e-03 |
| 43 | 3.693 | 4.265 | 0.572 | 1.033 | 6.652695e-05 |
| 44 | 3.614 | 4.287 | 0.673 | 1.135 | 2.321598e-05 |
| 45 | 3.431 | 4.153 | 0.722 | 1.064 | 2.157225e-06 |
| 46 | 3.764 | 4.263 | 0.498 | 1.098 | 8.500630e-04 |
| 47 | 3.623 | 4.162 | 0.539 | 1.094 | 3.311606e-04 |
| 48 | 3.469 | 4.204 | 0.734 | 1.047 | 1.099687e-06 |
| 49 | 3.601 | 4.002 | 0.401 | 1.336 | 2.366637e-02 |
| 50 | 3.652 | 4.245 | 0.593 | 1.185 | 2.698488e-04 |
| 51 | 3.608 | 4.204 | 0.596 | 1.183 | 2.494405e-04 |
| 52 | 3.846 | 4.321 | 0.476 | 1.323 | 7.174397e-03 |
| 53 | 3.572 | 4.117 | 0.545 | 1.110 | 3.446290e-04 |
| 54 | 3.533 | 4.511 | 0.978 | 0.981 | 1.574084e-10 |
| 55 | 3.637 | 4.181 | 0.544 | 1.363 | 3.052196e-03 |
| 56 | 3.470 | 4.147 | 0.677 | 1.056 | 6.109291e-06 |
| 57 | 3.631 | 4.206 | 0.575 | 1.088 | 1.300066e-04 |
| 58 | 3.626 | 4.214 | 0.587 | 1.163 | 2.387147e-04 |
| 59 | 3.592 | 4.359 | 0.766 | 1.255 | 1.441879e-05 |
| 60 | 3.459 | 4.306 | 0.847 | 1.086 | 1.111670e-07 |
| 61 | 3.586 | 4.636 | 1.050 | 1.384 | 2.070639e-07 |
| 62 | 3.647 | 4.437 | 0.790 | 1.176 | 2.597081e-06 |
| 63 | 3.634 | 4.139 | 0.505 | 1.157 | 1.291805e-03 |
| 64 | 3.444 | 4.135 | 0.691 | 1.349 | 2.005913e-04 |
| 65 | 3.533 | 4.310 | 0.777 | 1.045 | 3.184540e-07 |
| 66 | 3.564 | 4.285 | 0.721 | 1.042 | 1.425469e-06 |
| 67 | 3.667 | 4.283 | 0.616 | 0.984 | 9.377156e-06 |
| 68 | 3.415 | 4.263 | 0.848 | 1.114 | 1.880069e-07 |
| 69 | 3.535 | 4.055 | 0.520 | 1.070 | 3.867826e-04 |
| 70 | 3.567 | 4.230 | 0.662 | 1.087 | 1.508066e-05 |
| 71 | 3.563 | 4.238 | 0.675 | 1.060 | 7.027794e-06 |
| 72 | 3.629 | 4.324 | 0.694 | 1.075 | 5.403097e-06 |
| 73 | 3.654 | 4.206 | 0.553 | 1.156 | 4.728816e-04 |
| 74 | 3.529 | 4.322 | 0.793 | 1.124 | 9.885601e-07 |
| 75 | 3.559 | 4.145 | 0.586 | 1.068 | 7.792340e-05 |
| 76 | 3.597 | 4.182 | 0.586 | 1.250 | 5.977232e-04 |

For peer review only - http://bmjopen.bmj.com/site/about/guidelines.xhtml

```
77           3.627           4.365             0.738           1.083 1.969082e-06
78           3.518           4.126             0.608           0.963 8.083937e-06
79           3.521           4.233             0.712           1.208 2.599152e-05
80           3.610           4.279             0.670           0.923 5.504651e-07
81           3.534           4.138             0.604           1.080 5.789996e-05
82           3.599           4.161             0.562           1.150 3.621040e-04
83           3.619           4.208             0.589           1.457 2.695669e-03
84           3.553           4.270             0.717           1.248 3.889592e-05
85           3.647           4.381             0.734           1.346 8.421207e-05
86           3.525           4.242             0.718           0.994 6.041832e-07
87           3.590           4.230             0.640           0.943 2.132386e-06
88           3.505           4.099             0.594           1.061 5.770850e-05
89           3.469           4.248             0.779           1.038 2.615707e-07
90           3.619           4.077             0.458           1.260 6.644815e-03
91           3.571           4.133             0.561           1.146 3.526227e-04
92           3.704           4.201             0.497           0.922 9.906683e-05
93           3.722           4.488             0.766           1.278 1.969742e-05
94           3.576           4.155             0.579           1.188 3.696589e-04
95           3.577           4.250             0.674           1.322 2.128204e-04
96           3.489           4.272             0.783           1.122 1.218046e-06
97           3.590           4.259             0.670           1.001 2.797319e-06
98           3.529           4.385             0.856           0.906 7.912775e-10
99           3.664           4.403             0.738           1.124 3.974174e-06
100          3.679           4.304             0.626           1.048 2.117619e-05


Pooled results:
---------------
 AQLQ_BTBL_mean AQLQ_BTBL_sd AQLQ_FU12_mean AQLQ_FU12_sd AQLQ_change_mean AQLQ_change_sd    min_P_val max_P_val count_p_nonsig
          3.579        0.085          4.236         0.11            0.657          0.134 1.574084e-10   0.80313              1
---------------------------------------------------------------------------------------------------------------------------------

2. Multiple linear regression (MLR)
===================================
60 patients have a 12 month follow-up record and are included in the non-imputed/imputed analysis below

a) MLR performed using non-imputed data (using median values for centering):
============================================================================
BTBL n= 40 (mean AQLQ = 3.557 )
FU12 n= 37 (mean AQLQ = 4.243 )
paired n = 28
MLR modelled the outcome as the change in AQLQ from BT baseline to 12 month follow-up with covariates:
age at first BT procedure, BMI at BT baseline, FEV1 (% predicted) at BT baseline and eosinophil count (blood) at BT baseline

Call:
lm(formula = AQLQdiff ~ Adj_BT1_CALC_Age + Adj_BL_CALC_Bmi +
    Adj_BL_CALC_FEV1_Predicted + Adj_BL_EosinophilCount_blood,
    data = AQLQ_DIFF)

Residuals:
     Min       1Q   Median       3Q      Max
```

```
-1.31511 -0.31958 -0.02134  0.42481  1.09010

Coefficients:
                             Estimate Std. Error t value Pr(>|t|)    
(Intercept)                  0.787139   0.161073   4.887 0.000165 ***
Adj_BT1_CALC_Age            -0.063336   0.012883  -4.916 0.000155 ***
Adj_BL_CALC_Bmi             -0.049952   0.025250  -1.978 0.065374 .  
Adj_BL_CALC_FEV1_Predicted   0.013192   0.007707   1.712 0.106266    
Adj_BL_EosinophilCount_blood -0.038687   0.526264  -0.074 0.942309    
---
Signif. codes:  0 '***' 0.001 '**' 0.01 '*' 0.05 '.' 0.1 ' ' 1

Residual standard error: 0.6251 on 16 degrees of freedom
  (7 observations deleted due to missingness)
Multiple R-squared:  0.6721,    Adjusted R-squared:  0.5901 
F-statistic: 8.199 on 4 and 16 DF,  p-value: 0.0008522


b) MLR using MICE:
==================
                                est          se           t       df   Pr(>|t|)       lo 95       hi 95 nmis       fmi    lambda
(Intercept)             2.255356211 1.310516486  1.72096745 27.49264 0.09649391 -0.43134875 4.942061173   NA 0.4872430 0.4512508
BT1_CALC_Age           -0.028672937 0.015566560 -1.84195714 28.99557 0.07573107 -0.06051034 0.003164465    0 0.4597051 0.4236801
BL_CALC_BMI            -0.030466048 0.024170045 -1.26048783 29.27763 0.21744708 -0.07987899 0.018946892    2 0.4545732 0.4185448
BL_CALC_FEV1_Predicted  0.008620545 0.009267073  0.93023383 27.04739 0.36047888 -0.01039236 0.027633448    3 0.4954656 0.4594883
BL_EosinophilCount_blood -0.076979354 0.788169309 -0.09766855 26.05245 0.92294303 -1.69692585 1.542967139   17 0.5139486 0.4780146
```

For peer review only

BMJ Open

# Efficacy and safety of bronchial thermoplasty in clinical practice: a prospective, longitudinal, cohort study using evidence from the UK Severe Asthma Registry


| | |
|---|---|
| Journal: | *BMJ Open* |
| Manuscript ID | bmjopen-2018-026742.R1 |
| Article Type: | Research |
| Date Submitted by the Author: | 25-Mar-2019 |
| Complete List of Authors: | Burn, Julie; The Newcastle upon Tyne Hospitals NHS Foundation Trust, Northern Medical Physics and Clinical Engineering<br>Sims, Andrew; The Newcastle upon Tyne Hospitals NHS Foundation Trust, Northern Medical Physics and Clinical Engineering; University of Newcastle upon Tyne, Institute of Cellular Medicine<br>Patrick, Hannah; National Institute for Health and Care Excellence, Observational Data Unit<br>Heaney, Liam; Queens University Belfast, Centre for Experimental Medicine<br>Niven, Robert; Manchester Academic Health Science Centre; Manchester University NHS Foundation Trust |
| <b>Primary Subject Heading</b>: | Respiratory medicine |
| Secondary Subject Heading: | Respiratory medicine, Evidence based practice, Health informatics |
| Keywords: | Asthma < THORACIC MEDICINE, Bronchoscopy < THORACIC MEDICINE, Respiratory physiology < THORACIC MEDICINE |
| | |


SCHOLARONE™
Manuscripts

For peer review only - http://bmjopen.bmj.com/site/about/guidelines.xhtml

**TITLE**

Efficacy and safety of bronchial thermoplasty in clinical practice: a prospective, longitudinal, cohort study using evidence from the UK Severe Asthma Registry

**AUTHORS**

Burn J[1], Sims AJ[1,2], Patrick H[3], Heaney LG[4], Niven RM[5,6]

[1] The Newcastle upon Tyne Hospitals NHS Foundation Trust, UK, [2] Institute of Cellular Medicine, Faculty of Medical Sciences, University of Newcastle upon Tyne, UK, [3] National Institute for Health and Care Excellence, London, UK, [4] Centre for Experimental Medicine, Queen's University of Belfast, UK, [5] Manchester Academic Health Science Centre, Manchester University, UK, [6] Manchester University NHS Foundation Trust, UK.

**CORRESPONDING AUTHOR**

Julie Burn,
Northern Medical Physics and Clinical Engineering,
The Newcastle upon Tyne Hospitals NHS Foundation Trust,
Freeman Hospital,
Newcastle upon Tyne,
NE7 7DN,
UK
julie.burn@nuth.nhs.uk
+44 (0)191 233 6161

Andrew J Sims,
Northern Medical Physics and Clinical Engineering,
The Newcastle upon Tyne Hospitals NHS Foundation Trust,
Newcastle upon Tyne,
NE7 7DN,
UK
&
Institute of Cellular Medicine,
Faculty of Medical Sciences,
University of Newcastle upon Tyne,
Newcastle upon Tyne,
UK

Hannah Patrick,
Observational Data Unit,
National Institute for Health and Care Excellence,
London,
UK

Liam G Heaney,

For peer review only

Centre for Experimental Medicine,
Queen's University of Belfast,
Belfast,
UK

Robert M Niven,
Manchester Academic Health Science Centre,
Manchester University,
Manchester,
UK
&
The North West Lung Centre,
Manchester University NHS Foundation Trust,
Manchester,
UK

**KEYWORDS:** Asthma, Bronchoscopy, Respiratory physiology, Bronchial Thermoplasty, Observational Study, Registries

**WORD COUNT:** 4000

## ABSTRACT

*Objectives*: Use data from the UK Severe Asthma Registry (UKSAR) to assess the efficacy and safety of bronchial thermoplasty (BT) in routine UK clinical practice and to identify characteristics of 'responders'.

*Design*: Prospective, longitudinal, cohort, multi-centre registry study.

*Setting*: All (11) UK centres performing BT.

*Participants and intervention*: Patients receiving BT in the UK between 01/06/2011-30/09/2016 who had consented to data entry into UKSAR (n=133). Efficacy data was available for 86 patients with a BT baseline and at least one follow-up record. Safety data was available for 131 patients with at least one BT procedure record.

For peer review only

*Primary and secondary outcome measures*: Efficacy: AQLQ , ACQ, EuroQol, HADS anxiety and HADS depression scores, $FEV_1$ (% predicted), rescue steroid courses, unscheduled healthcare visits (A&E/Asthma clinic/GP), hospital admissions and days lost from work/school. Safety: peri-procedural events, device problems and any other safety-related findings. Responder analysis: differences in baseline characteristics of 'responders' (≥0.5 increase in AQLQ at 12 months) and 'non-responders'.

*Results*: Following Bonferroni correction for paired comparisons, mean improvement in AQLQ at 12 months follow-up compared with BT baseline was statistically and clinically significant (0.75, n=28, p=0.0003). Median reduction in hospital admissions/year after 24 months follow-up was also significant (-1.0, n=26, p<0.0001). No deterioration in $FEV_1$ was observed. From 28 patients with AQLQ data at BTBL and 12 month follow-up, there was some evidence that lower age may predict AQLQ improvement. 18.9% (70/370) of procedures and 44.5% (57/128) of patients were affected by an adverse event; only a minority were considered serious.

*Conclusions*: Improvement in AQLQ is consistent with similar findings from clinical trials. Other efficacy outcomes demonstrated improving trends without reaching statistical significance. Missing follow-up data impacted this study but multiple imputation confirmed observed AQLQ improvement. The safety review suggested BT is being performed safely in the UK.

**ARTICLE SUMMARY**

**Strengths and limitations of this study**

- The registry captured detailed morbidity and efficacy outcomes in the largest series of severe asthma patients having bronchial thermoplasty in routine UK clinical care, allowing comparison with outcomes reported from clinical trials.
- Comprehensive coverage of UK clinical practice, with almost 100% UK coverage of BT procedures being carried out post licence, thus avoiding selection bias.

For peer review only

- Improvement in AQLQ at 12 months assessed by paired analysis was confirmed with mixed effects modelling. Results from complete case analysis were confirmed with multiple imputation to account for the effect of missing data.
- Limitations include the lack of a comparator group and changes over time including prescribed drugs, comorbidities, weight and psychological factors which may affect the measured outcomes.

**INTRODUCTION**

Bronchial thermoplasty (BT) is an established non-pharmacological treatment for severe asthma. Thermal energy is applied to the airway wall to reduce bronchial smooth muscle, limiting its ability to contract. It is normally delivered in three bronchoscopic procedures, approximately 3-4 weeks apart. Clinical trials,[1-3] and follow-up studies,[4-6] provided early evidence of the efficacy and safety of BT but left uncertainty over whether the trial results would translate into clinical practice.

The need for more evidence from routine practice was identified by a Cochrane Review in 2014,[7], in guidelines jointly published by the European Respiratory Society and the American Thoracic Society,[8], and in the United Kingdom (UK) by the National Institute for Health and Care Excellence (NICE) in 2012. NICE reviewed BT,[9] and recommended collection of safety and efficacy outcomes through the UK Severe Asthma Registry (UKSAR),[10].

UKSAR was established in 2006 to collect data on patients with severe asthma and, in response to NICE's recommendations, was expanded to incorporate BT. A recent study of procedural and short-term safety of BT in UK clinical practice using both evidence from UKSAR and routine health data,[11] reported that although the rate of adverse events appeared higher than reported from clinical trials, only a minority were considered significant and that the higher rate may be explained by greater severity of asthma in patients being treated in this real world setting.

For peer review only

Several case series have been published, reporting on BT in clinical practice,[12-17]. These and an interim analysis from a large manufacturer-sponsored study,[18] all demonstrate some improvement in quality of life, reductions in exacerbations and health care resource utilisation and, in general concur with the findings of the clinical trials that BT is safe, with none raising any serious concerns. However, Thomson and Chanez,[19] recently reported on the contributions of the evidence to date in assessing the efficacy of BT in clinical practice and conclude that uncertainty remains.

The main aim of this study is to assess the longer term efficacy for BT in UK clinical practice. Secondary aims are to use experience from clinical practice to identify the characteristics of those patients most likely to benefit from BT, and to update safety evidence reported previously. It uses data collected in UKSAR and is one of the largest 'real-world' observational studies of BT to date, with all UK centres performing BT contributing data.

## METHODS

The study was designed to follow the recommendations of NICE Guidance,[9], and also current international efforts to improve data collection outside of a clinical trial setting for new technologies,[20,21].

### Registry design

Safety and efficacy outcomes and the proposed data fields to be added to the baseline, procedural and follow-up pages in UKSAR were agreed through consultation with clinicians in the UK Severe Asthma Network and with the support of NICE (Supplement 1, Table S1). Guidance on submitting data to UKSAR was issued to all centres providing BT to achieve consistent data capture. Modifications to UKSAR to include BT therapy were reported in Burn et al,[11].

### Inclusion criteria

For peer review only

Eligible patients were those selected to receive BT in the UK between 01/06/2011 and 30/09/2016 with data in UKSAR. Patients included in this study must have received BT treatment, had a valid BT baseline (BTBL) record and at least one follow-up record.

**Patient and Public Involvement**

Patients and the public were not involved in the design of this study. However, the findings are presented as evidence in the update of NICE interventional guidance on Bronchial Thermoplasty,[9].

**Registry coverage**

During the data collection period regular contact was maintained with the sole manufacturer of the BT device to verify that all (11) UK centres performing BT were entering data into UKSAR. Regular contact with participating centres was maintained to encourage data entry and to confirm procedure numbers.

**Data extraction**

Ethics approval for the registry was provided by the Office for Research Ethics Committees Northern Ireland (10/NIR02/37). Patients having BT between June 2011 and September 2016 were invited to give fully informed written consent to record r information  on UKSAR. BTBL, procedure and follow-up records in UKSAR at 31st October 2016 were extracted for analysis. In accordance with registry information governance requirements the data were provided for analysis in anonymised form.

**Data cleaning**

The follow-ups nearest in time to 6, 12, 24, 36, 48 and 60 months following the last BT procedure were allocated to these follow-up points and the median times to these were calculated. Count data for rescue steroid courses, unscheduled healthcare visits, hospital admissions and days lost from work or school were annualised to compare rates before and after BT.

**Patient baseline characteristics**

Patient baseline characteristics were obtained from UKSAR including: age at first BT procedure, gender, Body Mass Index (BMI), smoking status, eosinophil count (blood), asthma status (including pre-bronchodilator forced expiratory volume in 1 second ($FEV_1$) (% predicted) and Asthma Quality of

For peer review only - http://bmjopen.bmj.com/site/about/guidelines.xhtml

Life Questionnaire (AQLQ), 32 question score,[22]), Asthma Control Questionnaire (ACQ) score, EQ-5D-3L descriptive index score, HADS anxiety and depression scores, rescue steroid courses, unscheduled healthcare usage, hospital admissions and days lost from work/school in the previous year, maintenance oral steroids (Y/N), oral steroid dose, anti-IgE medication (Y/N). Characteristics of age, gender, $FEV_1$ and AQLQ score were compared with patients enrolled in clinical trials,[1-3] and characteristics of those included in the efficacy study were compared with those excluded to check for exclusion bias.

**Efficacy outcomes**

The efficacy outcomes considered were AQLQ , ACQ, EQ-5D-3L (descriptive index), HADS anxiety and HADS depression scores, $FEV_1$ (% predicted), rescue steroid courses, unscheduled healthcare visits (A&E/Asthma clinic/GP), hospital admissions and days lost from work/school.

Outcome measures at BTBL and follow-up points were calculated using all available data and compared pair-wise between BTBL and both 12 and 24 month follow-ups.

**Potential confounding factors**

Types and doses of medications at BTBL and follow-up were not included as outcome measures but were studied as potential confounding factors. For the 86 patients in the efficacy study, the proportions taking maintenance oral steroids and the median doses at BTBL and 12 month follow-up were compared. The numbers of patients who either remained on oral steroids, off oral steroids or started/stopped oral steroids between BTBL and 12 month follow-up were also calculated.

A biologic therapy (Omalizumab) may also have been prescribed as an anti-IgE medication, and the proportions of patients for whom the anti-IgE registry field was ticked at BTBL and 12 month follow-up were compared.

**Statistical analysis**

For peer review only

Mean values were calculated for continuous variables and median values for count data. Paired comparisons were performed using: two-sided paired t-tests for continuous variables; two-sided single sample bootstrap hypothesis tests for count data, with data grouped by follow-up time points. All variables were checked for normality using Shapiro-Wilk tests, and p values obtained by t-tests were confirmed using two-sided non-parametric bootstrap tests. Comparison of groups used two sample t-tests for continuous variables and Wilcoxon rank sum test for count data. Comparisons of proportions used proportion test and fisher test. The Bonferroni method was used to adjust for multiple comparisons. Analysis was performed using the 'R' statistical programming language with a significance level of 95%.

**Missing data and additional statistical analyses**

To assess the effect of missing data, and potential bias due to using available case analysis, additional statistical analyses were performed.

(i) Multiple imputation was applied to study the change in AQLQ from BTBL to 12 month follow-up. The R package 'MICE',[23] was used to impute missing data values in the records of the 60 patients having both BTBL and 12 month follow-up records. The levels of missing data were examined and the number of imputations used in the model set to 100 (Supplement 1, Table S2),[24-27].

Paired t-tests were undertaken on each of the 100 imputed datasets from which the mean and standard deviation of the 'mean change in AQLQ' were calculated. The rate of 'mean change in AQLQ' < 0.5 (not clinically significant) and the rate of non-significant p-value (< 0.05) from the 100 imputations were also reported.

(ii) A linear mixed effects model was used to measure the change in AQLQ following BT by using all available observations without imputation with the R package 'lmer',[28]. This class of model accounts for repeated observations (multiple follow-ups), tolerates missing values and estimates sizes of fixed and random effects separately. The model response was AQLQ. There was one fixed effect – time, to test whether AQLQ changed over time; and two random effects: of patients on

For peer review only

intercept (i.e. it accounted for different baseline AQLQ between patients), and of patients on slope (i.e. it accounted for different patients having different rates of change of AQLQ after treatment). The null hypothesis of there being no change in AQLQ over time was tested using a parametric bootstrap hypothesis test with a generalised likelihood ratio test statistic. Confidence intervals were estimated using bootstrapping.

**Comparison of responders with non-responders**

Using the AQLQ Minimally Important Clinical Difference (MCID), a responder was defined as ≥0.5 increase in AQLQ score at 12 month follow-up compared with BTBL. The baseline characteristics of responders were compared with non-responders to assess potential characteristics of those most likely to benefit from BT.

A linear model was constructed with change in AQLQ (12 month follow-up – BTBL) as the response variable and four covariates: BT1 age, baseline BMI, baseline $FEV_1$ (% predicted), baseline eosinophil count (blood). Patients with AQLQ observations at BTBL and 12 month follow-up were included. All covariates were centred on their median values.

To assess the effect of missing covariates, the model was repeated using multiple imputation with the records of 60 patients having BTBL and 12 month follow-up records. Median centering was not possible within the multiple imputation model.

**Safety outcomes**

UKSAR records added since a previous report of safety outcomes,[11] were reviewed and peri-procedural events, device problems and other potential safety findings were tabulated.

**RESULTS**

**Data and study participants**

Eighty six patients who received BT treatment, had a valid BTBL record and at least one follow-up record in UKSAR, were eligible for inclusion in the efficacy analysis (Fig. 1). The total follow-up time

For peer review only

in person-years from final BT treatment until the data extraction date of 31st October 2016 was 253.73 (median 2.85). Data in UKSAR for 128 patients having at least one BT procedure were available for review of safety outcomes. The total numbers of BT records available in UKSAR are shown in Supplement 1, Table S3.

**Baseline characteristics**

126 patients had a valid BTBL record (female: 69.8%; mean age at first BT: 43.7 ± 12.2 years (n=125); BMI: 31.8 ± 7.5 kg/m$^2$ (n=119); Non-smoker/Ex -smoker: 98.3% (n=117); $FEV_1$ (% predicted): 71.0 ± 21.8 (n=118)).

The baseline characteristics of patients included in the efficacy study (n=86) and excluded (n=40) are shown in Supplement 1 (Table S4). The mean age of those included was lower than the excluded group (not significant after Bonferroni correction). No other significant differences were found between the 2 groups.

Compared with published clinical trials,[1-3], patients in this efficacy study were on average older, with lower $FEV_1$ (% predicted) values (except for the RISA trial) and lower AQLQ scores (Table 1).

**Table 1: Baseline characteristics of BT patients in UKSAR compared with clinical trials**

| | UKSAR | AIR2 trial | AIR trial | RISA trial |
|---|---|---|---|---|
| **Mean age at BT1 (years)** | 43.7 ± 12.2 (n=125)<br>Range 21-74 | 40.7 ± 11.9 (†BT, n=190)<br>40.6 ± 11.9 (††C, n=98) | 39.4 ± 11.2 (BT, n=55)<br>41.7 ± 11.4 (C, n=54) | 39.1 ± 13.0 (BT, n=15)<br>42.1 ± 12.6 (C, n=17) |
| **% female** | 70 (n=126) | 57 (BT), 61 (C) | 56 (BT), 57 (C) | 60 (BT), 41(C) |
| **Pre-bronch $FEV_1$ (% predicted)** | 71.0 ± 21.8 (n=118)<br>Range: 18-109 | 77.8 ± 15.7 (BT)<br>79.7 ± 15.1 (C) | 72.7 ± 10.4 (BT)<br>76.1 ± 9.3 (C) | 62.9 ± 12.2 (BT)<br>66.4 ± 17.8 (C) |
| **AQLQ score†††** | 3.66 ± 1.35 (n=81)<br>Range 1.0 - 6.81 | 4.30 ± 1.17 (BT)<br>4.32 ± 1.21 (C) | 5.6 ± 1.1 (BT)<br>5.7 ± 0.9 (C) | 3.96 ± 1.34 (BT)<br>4.72 ± 1.06 (C) |

† BT=subjects receiving BT treatment
†† C=control subjects
†††A higher AQLQ score represents better quality of life

**Efficacy outcomes**

Efficacy outcome measures at BTBL, 12 and 24 month follow-up points are shown in Table 2 (see Supplement 1, Table S5 for data at 6, 36, 48 and 60 months and Figure S1). There was a clinically significant (>=0.5) improvement in AQLQ after 12 months. There were improvements in depression scores at 12 months, reduction in health care resource utilisations and hospitalisations at 12 and 24 months and steroid courses were reduced at 12 months and more than halved at 24 months. After Bonferroni correction, only improvement in AQLQ after 12 months and reduction in hospital admissions at 24 months were statistically significant.

**Table 2: Efficacy: all available data in UKSAR at BTBL, 12 and 24 month follow-up; paired comparisons of BTBL data with outcomes at 12 and 24 month follow-up (data shown as mean (sd) or median [LQ - UQ])**

| | BTBL (n=86) | FU12 (n=60) | FU24 (n=34) | BTBL to FU12 (paired) | BTBL to FU24 (paired) |
|---|---|---|---|---|---|
| **AQLQ score** | 3.64 (1.26) n=59 | 4.24 (1.45) n=37 | 4.40 (1.62) n=19 | 0.75 n=28 (p=0.0003)[†] | 0.39 n=16 (p=0.148) |
| **EQ-5D score** | 0.53 (0.38) n=42 | 0.62 (0.38) n=29 | 0.65 (0.35) n=18 | 0.008 n=18 (p=0.909) | 0.029 n=13 (p=0.706) |
| **ACQ score** | 3.28 (1.36) n=49 | 2.75 (1.34) n=40 | 3.06 (1.27) n=21 | -0.43 n=36 (p=0.083) | -0.26 n=19 (p=0.370) |
| **HADS score (Anxiety)** | 8.52 (5.54) n=48 | 6.46 (5.20) n=28 | 5.28 (5.65) n=18 | -1.60 n=20 (p=0.078) | -0.93 n=14 (p=0.216) |
| **HADS score (Depression)** | 6.46 (5.25) n=48 | 5.07 (4.50) n=28 | 4.67 (4.85) n=18 | -1.60 n=20 (p=0.047) | -0.57 n=14 (p=0.336) |
| **FEV1 (% predicted)** | 69.65 (21.71) n=82 | 74.90 (21.34) n=52 | 72.71 (21.08) n=31 | 3.51 n=49 (p=0.152) | 2.57 n=30 (p=0.560) |
| | | | | | |
| **Days from last procedure to follow-up** | - | 381.5 [363.8 - 408] n=60 | 738.5 [720.3 - 775.8] n=34 | - | - |
| **Rescue steroid courses (annualised)** | 4 [2 - 5.5] n=75 | 3.1 [1.4 - 5.8] n=55 | 1.9 [0.8 - 4.9] (n=29) | -0.26 n=49 (p=0.151) | -1.42 n=27 (p=0.129) |
| **Unscheduled healthcare[††] (annualised)** | 5 [2 - 6] n=71 | 3.2 [1.1 - 5.6] n=52 | 1.3 [0 - 2.9] (n=29) | -0.93 n=47 (p=0.018) | -1.55 n=24 (p=0.031) |

| | | | | | |
|---|---|---|---|---|---|
| **Hospital admissions (annualised)** | 2<br>[0 - 3]<br>n=76 | 0<br>[0 - 1.9]<br>n=55 | 0<br>[0 - 0.6]<br>n=29 | -2.0<br>n=51 (p=0.050) | -1.0<br>n=26 (p<0.0001)† |

† Significant with Bonferroni correction applied for 9 paired comparisons (p < 0.006) at each follow-up point

†† Includes visits to A&E, GP and asthma clinic

Table 3 shows results of paired t-tests following multiple imputation analysis for AQLQ score at 12 months compared with BTBL. 99/100 imputations demonstrated a statistically significant mean increase in AQLQ at 12 months follow-up; 92/100 imputations demonstrated a clinically significant mean increase in AQLQ (>=0.5).

**Table 3: Multiple Imputation (MI): Paired comparisons of AQLQ at BTBL and 12 month follow-up (FU12) compared with non-imputed data**

| | AQLQ score (BTBL) | AQLQ score (FU12) | AQLQ change | P value |
|---|---|---|---|---|
| † Non-imputed data (n=28, mean ± SEM) | 3.52 ± 0.21 | 4.27 ± 0.27 | 0.75 ± 0.07 | 0.0003 |
| †† MI data (n=60) | 3.58<br>± 0.09 | 4.24<br>± 0.11 | 0.66<br>± 0.13††† | < 0.05 †††† |

† Paired t-test used to compare 28 patients who had AQLQ measurements at BTBL and FU12

†† 60 patients had BTBL and FU12 records which could be used in the multiple imputation. 100 imputations were performed, resulting in 100 values for mean AQLQ score at BTBL and 12 month follow-up, and mean change in AQLQ. The means of these values are presented in the table. Columns included in the imputation: Hospital, Gender, BT1 Age, BL BMI, BL Smoking Status, BL Eosinophil Count (blood), BL FEV1 (% predicted), BL AQLQ, BL Eq_5d, BL ACQ, BL HADS (Anxiety), BL HADS (Depression), BL Rescue Steroid Courses, BL Unscheduled Healthcare Visits, BL Hospital Admissions, FU12 Age, FU12 BMI, FU12 Smoking Status, FU12 Eosinophil Count (blood), FU12 FEV1 (% predicted), FU12 AQLQ, FU12 Eq_5d, FU12 ACQ, FU12 HADS (Anxiety), FU12 HADS (Depression), FU12 Rescue Steroids (annualised), FU12 Unscheduled Healthcare Visits (annualised), FU12 Hospital Admissions (annualised)

††† 92/100 imputed datasets had a mean AQLQ change >= 0.5

†††† 99/100 imputed datasets had p < 0.05

Fig. 2 shows the AQLQ repeated measures model for patients with AQLQ score at BTBL and at least one follow-up. From 128 AQLQ observations for 42 patients (median follow-up: 572.4 days), values

For peer review only

of the fixed effects were 3.93 (95%CI 3.55 to 4.43) for baseline AQLQ and +0.19 (95% CI 0.00 to 0.36) AQLQ/year for the mean annual change of AQLQ after treatment. The random effect of baseline AQLQ due to individual patients was 1.06 (0.75 to 1.37), the random effect of individual patients on the annual change of AQLQ was 0.15 (0.02 to 0.39) AQLQ/year, and the residual error standard deviation in the model was 0.81 (0.67 to 0.92) AQLQ. The null hypothesis, of no change in AQLQ over time, was rejected at the 5% level (p=0.027). Responders (coloured blue) are those with an >= 0.5 increase in AQLQ at 12 month follow-up compared with BTBL.

For peer review only

There was no significant change in mean values of $FEV_1$ (% predicted) at 12 or 24 months.

The large number of missing data points and zero entries for 'days lost from work/school' prevented reliable analysis. Table 4 shows all available data for days lost from work or school at BTBL (assumed to represent the preceding 12 months) and 12 month follow-up (annualised). Prior to BT, 45.2% of patients (19/42) reported losing at least 1 day per year, compared with 13.5% (5/37) in the year following BT; p = 0.005 by proportion test.

**Table 4: Data in UKSAR for days lost from work/school (self-reported) at BTBL and 12 month follow-up**

| **Days lost from work/school data** | **BTBL†** | **FU12††** |
|---|---|---|
| Number of patients with records | 86 | 60 |
| Number of patients with days lost reported | 42 | 37 |
| - Patients reporting zero days | - 23 | - 32 |
| - Patients reporting > zero days | - 19 (45.2%) | - 5 (13.5%) |
| Number of days lost reported | 406 | 61 |
| - Median [IQR] days lost/person (for all patients reporting days lost) | - 0 [0-10] | - 0 [0-0] |
| - Median [IQR] days lost/person (for patients reporting > zero days lost) | - 10 [7-15] | - 13.0 [8.9-15.1] |

† Data at BTBL is assumed to represent a 12 month period
†† Data at FU12 is annualised

**Potential confounding factors**

For 86 patients in the efficacy study, the proportion taking maintenance oral steroids at BTBL was 55/85 (64.7%) compared with 36/60 (60%) at 12 month follow-up; p=0.56. The proportion for whom

the anti-IgE registry field was ticked (indicating a biologic) at BTBL was 14/84 (16.7%) compared with 9/54 (16.7%) at 12 month follow-up; p=1.0.

34 patients took maintenance oral steroids at both BTBL and 12 month follow-up, 20 patients did not take them at either BTBL or 12 month follow-up, 4 patients took them at BTBL but not 12 month follow-up, 2 patients did not take them at BTBL but did at 12 month follow-up. For 31/34 patients taking maintenance oral steroids at BTBL and 12 month follow-up (with valid dose information), the median dose (mg/day) at BTBL was 25 [IQR:15-30] compared with 20 [IQR:10-32.5] at 12 month follow-up; p=0.44.**Analysis of responders versus non-responders**

18/28 patients with AQLQ data at BTBL and 12 month follow-up were classified as responders (defined by >=0.5 increase in AQLQ); 10/28 patients were classified as non-responders. The baseline characteristics of both groups are shown in Table 5.

**Table 5: Responder analysis: baseline characteristics of patients who had AQLQ data at BT baseline and 12 month follow-up (a responder is defined here as having >= 0.5 increase in AQLQ at 12 month follow-up compared with BT baseline). Data shown as mean (sd) or median [min,LQ,UQ,max].**

| Characteristic | Responders (n=18) | Non-responders (n=10) |
|---|---|---|
| Age at first BT (years) | 35.78 (8.7) n=18 | 50.4 (11.0) n=10 |
| Female (%) | 61.1 n=18 | 60.0 n=10 |
| BMI (kg/m$^2$) | 27.7 (5.2) n=17 | 33.23 (10.7) n=10 |
| Non-smoker/Ex-smoker (%) | 100.0 n=17 | 88.9 n=9 |
| $FEV_1$ (% predicted) | 65.9 (22.1) n=18 | 62.3 (19.1) n=10 |
| Eosinophil count (blood) | 0.29 (0.25) n=13 | 0.51 (0.39) n=8 |
| AQLQ score | 3.48 (1.19) n=18 | 3.59 (1.02) n=10 |
| EQ-5D score | 0.54 (0.42) n=11 | 0.58 (0.39) n=9 |
| ACQ score | 3.59 (1.25) n=14 | 3.07 (0.71) n=9 |
| HADS score (Anxiety) | 6.20 (5.53) n=15 | 10.33 (5.29) n=9 |
| HADS score (Depression) | 4.53 (5.40) n=15 | 7.78 (5.04) n=9 |

For peer review only

| | | |
|---|---|---|
| **Rescue steroid courses (previous year)** | 5 [0,2,6,12] n=17 | 2 [0,1.25,3,7] n=10 |
| **Unscheduled healthcare† (previous year)** | 4.0 [0,1,6,12] n=16 | 3 [0,2,5,10] n=9 |
| **Hospital admissions (previous year)** | 1.5 [0,0,3.25,10] n=16 | 0 [0,0,1.5,4] n=10 |
| **Days lost from work or school (previous year)** | 0 [0,1,5,15] n=9 | 3.5 [0,0,9,15] n=4 |

† Includes visits to A&E, GP and asthma clinic

As reported in the pivotal AIR2 trial,[1], responders in this study also had lower mean baseline AQLQ and higher mean baseline ACQ than non-responders, however multivariate analysis showed that the only significant predictor of outcome was age at first BT procedure, whereby younger patients were more likely to have a greater improvement in AQLQ at 12 month follow-up (Supplement 2).

Multiple imputation was performed for the 60 patients having records at BTBL and 12 month follow-up. However, multivariate analysis using the pooled imputed datasets just failed to confirm age (p=0.076) as a significant predictor of improvement in AQLQ at 12 month follow-up (Supplement 2).

**Safety outcomes**

There were 370 procedure records in UKSAR, for 128 patients. Table 6 summarises peri-procedural events, device problems and potential safety-related events found following manual review of all procedure and follow-up records. Overall, 18.9% of procedures and 44.5% of patients were affected by an adverse event.

For peer review only

**Table 6: Safety**

| Peri-procedural events | Number of procedures affected (n=370†) | Number of patients affected (n=128††) |
|---|---|---|

| | | |
|---|---|---|
| Events preventing treatment completion (Excessive cough, discomfort and pain/bronchospasm) | 5 | 5 |
| Infection | 8 | 8 |
| Exacerbation | 13 | 11 |
| Asthma-related symptoms (Drop in FEV1, wheeze, shortness of breath, low Sao2) | 24 | 19 |
| Procedure related symptoms (Bronchospasm, dry cough, chest twinges/tightness/discomfort/pain) | 20 | 16 |
| Other (Left rib fracture) | 1 | 1 |
| Other (Metabolic acidosis) | 1 | 1 |
| Other (Inflamed airways, bleeding medial basal) | 1 | 1 |
| Other (Lung collapse, one slight) | 4 | 4 |
| Other (Procedure-related bradycardia) | 1 | 1 |
| **Additional reports** | | |
| Device-related (catheters needed replacement) | 2 | 2 |
| Prolonged stay (>=7 days) with no reason given) | 3 | 2 |
| A & E attendance with no details given | 1 | 1 |
| Airway tracheomalacia reported | 2 | 2 |
| BT3 postponed 2 months due to inflamed airways & pain | 1 | 1 |
| Three procedures not able to be performed: ††† | | |
| • BT1 only | - | 2 |
| • BT1 and BT2 only | - | 2 |
| **CT scan reports (up to 6 month follow-up)** (From 24 CT scan reports in 21 patients) | | |
| Central bronchiectasis | - | 1 |
| Other bronchiectasis | - | 2 |
| **Total events** | 87 | - |
| **Unique procedures/patients affected** | 70 (18.9%) | 57 (44.5%) |

† 370 procedures in total were entered with a date in UKSAR, but some had minimal procedural information recorded
†† Three patients were excluded as they had no procedure records entered in UKSAR
††† In addition, 4 patients hadn't completed all 3 procedures at 30/09/2016

Some peri-procedural adverse events, although reported in the 'Unanticipated procedural morbidity' field, could be classed as anticipated. Only a minority of procedures were affected by more serious adverse events; inflamed airways/bleeding (1), significant desaturation (1), lung collapse (one slight) (4), left rib fracture (1), metabolic acidosis/lactic acidosis (1), procedure-related bradycardia (1), procedure being stopped/early termination (5).

For peer review only - http://bmjopen.bmj.com/site/about/guidelines.xhtml

Nine procedures in 8 patients were carried out using general anaesthesia. The median number of activations for all procedures was 34 [min,LQ,UQ,max: 2,25,50.75,115]. For the procedures carried out under general anaesthesia the median number of activations was 38 [min,LQ,UQ,max: 12,30,39,50].

There were two recorded device problems relating to catheters; one was changed after 2 activations when a spark was noticed, in another kinking and infolding was reported after treatment of the right upper lobe.

**DISCUSSION**

In this 'real-world' study of BT, we used clinical registry data collected during routine UK clinical practice to analyse efficacy and safety outcomes. All UK centres performing BT were included and patients in this study were not subject to any specific exclusion criteria; their suitability for BT was determined on an individual basis by a multi-disciplinary team. Compared with three clinical trials, patients selected to receive BT in clinical practice were on average older, had lower baseline $FEV_1$ (except for RISA trial) and lower AQLQ scores.

The primary outcome for the AIR2 trial was AQLQ at 12 months and it showed an improvement in AQLQ of borderline statistical and below clinical significance compared with sham. The size of the sham placebo response was unprecedented and made interpretation of the findings complex. In our study, for 28 patients having AQLQ data at BTBL and 12 month follow-up, there was a statistically and clinically significant mean improvement in AQLQ at 12 month follow-up compared with BTBL. The reliability of the AIR2 trial reported improvement in AQLQ was questioned,[29-31] therefore we sought further assurance of our results by conducting multiple imputation analysis for 60 patients having both BTBL and 12 month follow-up records. This also showed a statistically and clinically significant mean improvement in AQLQ at 12 month follow-up, consistent with the non-imputed data.

For peer review only

The AQLQ MCID of 0.5 was originally calculated for patients with mild asthma and in a severe asthma population, may underestimate responders. It is assumed that MCID remains the same over the whole range of the scale however patients with lower baseline AQLQ score may perceive an increase of less than 0.5 as meaningful compared with patients starting with a higher score. Using 0.5 as the MCID, we still found a clinically significant mean improvement in AQLQ at 12 month follow-up compared with BTBL.

Use of repeated measures analysis for 42 patients having AQLQ data at BTBL and at least one follow-up addresses, in part, the arbitrary choice of 12 months for conventional paired analysis of change in AQLQ. This approach estimated that AQLQ increased by a mean of +0.19 /year (which was significantly greater than zero) after treatment, assuming it increased linearly. The improvement at 1 year predicted by the mean rate (0.19), taking into account all follow-up observations, was less than the observed paired improvement at 12 months (0.75). This suggests that there is still uncertainty over the characteristics of the long-term persistence effect of BT beyond 12 months.

For other efficacy outcomes, the data showed trends for improvement of similar magnitude to the clinical trials however the only other statistically significant change after Bonferroni correction was the median reduction in the annual rate of hospital admissions after 24 months. The data indicate a possible reduction in days lost from work/school but we report improvements with caution as the data includes many zero entries.

Although no significant improvement in $FEV_1$ (% predicted) was shown at any follow-up point, a small increase in absolute $FEV_1$ was seen with no subsequent decline, which is consistent with other reported studies.

Median steroid dose before and after BT was not defined as a primary efficacy outcome in our study but is a potential confounder. We found no-significant change in median oral steroid dose at 12 month follow-up compared with BTBL for those patients taking oral steroids at both time points;

For peer review only

however caution is advised as patients are prescribed a variety of medications. Likewise, biologics may confound outcomes, but only 16.7% of patients in the efficacy study were noted as being on anti-IgE medication, with the proportion staying constant between BTBL and 12 month follow-up.

An exploratory responder analysis with 4 covariates (no interactions) was used to identify patients who might potentially benefit from BT, comparing 18 responders with 10 non-responders. This showed interesting trends, but only younger age was a significant predictor of improvement in AQLQ. Multiple imputation failed to reproduce this finding.

Peri-procedural and short-term safety outcomes were previously reported using data from UKSAR and HES,[11]. It was found that 20.4% of procedures in the cohort were affected by at least one reported event (including procedural complication, emergency respiratory readmission or respiratory A&E attendance (without subsequent hospital admission) within 30 days).

This study sought to provide an update using the data available in UKSAR. We found that 18.9% of procedures and 44.5% of patients were affected by at least one event that could be identified from procedure or follow-up records. These rates were calculated using all 370 procedure records with at least the date field completed. However, some of these had no further details of the procedure so their effect on the rates is unknown. Conversely, many peri-procedural adverse events, although reported in the 'Unanticipated procedural morbidity' field, could have been classed as anticipated. Including only the more serious or genuinely unanticipated events in the safety analysis would decrease the rate of adverse events. Hence full details of all events were included to enable readers to judge the number and severity of events.

A strength of this study is the comprehensive coverage reported from clinical practice. We achieved almost 100% UK coverage of BT procedures being carried out post licence as confirmed by the sole manufacturer of the BT device. Data was obtained from centres performing lower numbers of

For peer review only

procedures and those that had previously participated in the clinical trials, thus avoiding any selection bias.

We used more than one statistical method to analyse the change in AQLQ, and the results were consistent in each.

A limitation of this study is the lack of comparator group so the observed improvements may be due to placebo effect. Additionally, over time, various factors affect the measured outcomes; patients are prescribed different drugs after BT and comorbidities, weight change, and psychological factors may all affect outcomes. We did not study paired data beyond 24 month follow-up as there were diminishing numbers of follow-up records available for analysis and other confounding factors may become more significant.

Despite the recommendation in NICE Guidance for data collection, the biggest limitation is the poor follow-up data completion, resulting in only a small number of patients with datasets available for follow-up analysis. Whilst we recognise that missing data limits generalisability, we performed multiple imputation analysis to address this and the imputation model did support the observed improvement in AQLQ score at 12 month follow-up. Improvements in several other outcome measures which were non-significant after correcting for multiple testing suggest that there is still a need to capture more longer-term outcome information to fully understand the effectiveness of BT.

**CONCLUSIONS**

In summary, this paper presents outcomes from the largest UK 'real-world' study of bronchial thermoplasty to date. The mean improvement in AQLQ at 12 months compared with BTBL is consistent with similar findings from clinical trials. No deterioration in $FEV_1$ (% predicted) was observed following BT and there was a significant reduction in hospital admissions at 24 month follow-up. However, improvements were not seen in all patients and an exploration of the characteristics of ‘responders’ to BT could only identify age as a possible predictor of outcome.

For peer review only

Current and future studies looking into factors which determine those who respond well to BT will be crucial to informing patient selection. Continued data collection would also help to understand whether the improvement in AQLQ observed for some patients is maintained over a longer period. Although a health economic analysis was beyond the scope of this study, the improvement in QoL and reduction in health care usage would suggest that BT may bring cost savings.

Of the adverse events recorded, only a minority were considered significant by the treating clinician. It appears that BT is being carried out safely in the UK, however long-term safety should continue to be monitored and the decision to select a patient for BT should continue to lie with a multi-disciplinary team who can assess their history and suitability for the procedure.

**ACKNOWLEDGEMENTS**

We are grateful for the support of staff at centres performing bronchial thermoplasty and completing data entry to the UK Severe Asthma Registry: Royal Brompton Hospital (London), Manchester University NHS Foundation Trust, Gartnavel General Hospital (Glasgow), Freeman Hospital (Newcastle upon Tyne), Heartlands Hospital (Birmingham), University Hospitals of Leicester, University Hospital Southampton, Aintree University Hospital (Liverpool), Queen Alexandra Hospital (Portsmouth), Addenbrooke's Hospital (Cambridge), Nottingham City Hospital. We are also grateful for the support of Sally Welham (British Thoracic Society) who provided invaluable advice to the steering group.

**CONTRIBUTORS**

LGH is founder and co-ordinator of the UK Severe Asthma Registry Data and provided data extracts. JB, AJS, HP, LGH and RMN contributed equally to the design of the study. JB and AJS were responsible for analysis of the data. JB, AJS, HP, LGH and RMN were responsible for interpretation of the analysis. JB, AJS, HP, LGH and RMN drafted, revised and approved the final version of the manuscript.

For peer review only

**COMPETING INTERESTS**

LGH has received grant funding from Medimmune, Novartis UK, Roche/Genentech Inc, Astra Zeneca and Glaxo Smith Kline, have taken part in Advisory Boards and given lectures at meetings supported by Glaxo Smith Kline, Respivert, Merck Sharpe & Dohme, Nycomed, Boehringer Ingelheim, Novartis and Astra Zeneca. LGH has received support funding to attend International Respiratory meetings (Astra Zeneca, Chiesi, Novartis, Boehringer Ingelheim and Glaxo Smith Kline) and has taken part in asthma clinical trials (GSK, Schering Plough, Synairgen and Roche/Genentech) for which his Institution was remunerated. LGH is Academic Lead for the Medical Research Council Stratified Medicine UK Consortium in Severe Asthma which involves Industrial Partnerships with Amgen, Johnson & Johnson, Genentech / Roche, Astra Zeneca / Medimmune, Aerocrine and Vitalograph. RN was PI on several of the thermoplasty trials, and has received honoraria for lecturing & attending advisory boards from Boston Scientific.

**FUNDING**

Pilot funding for the UK Severe Asthma Registry was provided as unrestricted research grants from Astra Zeneca, GlaxoSmithKline, Novartis and Medimmune.

The extension of the UK Severe Asthma Registry to include bronchial thermoplasty data collection was funded by NICE. JB and AJS are employed by The Newcastle upon Tyne Hospitals NHS Foundation Trust which hosts an External Assessment Centre funded by NICE.

**ETHICAL APPROVAL**

Ethics approval for the UK Severe Asthma Registry (UKSAR) was provided by the Office for Research Ethics Committees Northern Ireland (10/NIR02/37).

**PATIENT CONSENT**

Patients having BT between June 2011 and September 2016 were invited to give fully informed written consent for their information to be put on UKSAR.

For peer review only

**DATA SHARING**

The registry data used in this study were provided to The Newcastle upon Tyne Hospitals NHS Foundation Trust following approval by the Registry steering committee. In accordance with registry information governance requirements, the data were provided for analysis in anonymised form. The authors are not permitted to make the data publicly available as there is a risk that patients could be identified due to the small numbers involved.

**REFERENCES**


1. Castro M, Rubin AS, Laviolette M, Fiterman J, De Andrade Lima M, Shah PL, et al. Effectiveness and safety of bronchial thermoplasty in the treatment of severe asthma: a multicenter, randomized, double-blind, sham-controlled clinical trial. *Am J Respir Crit Care Med*. 2010;181(2):116-24.
2. Cox G, Thomson NC, Rubin AS, Niven RM, Corris PA, Siersted HC, et al. Asthma control during the year after bronchial thermoplasty. *N Engl J Med.* 2007;356:1327-37.
3. Pavord ID, Cox G, Thomson NC, Rubin AS, Corris PA, Niven RM, et al. Safety and efficacy of bronchial thermoplasty in symptomatic, severe asthma. *Am J Respir Crit Care Med*. 2007;176(12):1185-91.
4. Wechsler ME, Laviolette M, Rubin AS, Fiterman J, Lapa e Silva JR, Shah PL, et al. Bronchial thermoplasty: Long-term safety and effectiveness in patients with severe persistent asthma. *J Allergy Clin Immunol.* 2013;132(6):1295-302.
5. Thomson NC, Rubin AS, Niven RM, Corris PA, Siersted HC, Olivenstein R, et al. Long-term (5 year) safety of bronchial thermoplasty: Asthma Intervention Research (AIR) trial. *BMC Pulm Med*. 2011;11:8.
6. Pavord ID, Thomson NC, Niven RM, Corris PA, Chung KF, Cox G, et al. Safety of bronchial thermoplasty in patients with severe refractory asthma. *Ann Allergy Asthma Immunol*. 2013;111(5):402-7.


For peer review only

7. Torrego A, Solà I, Munoz AM, Roqué i Figuls M, Yepes-Nuñez JJ, Alonso-Coello P, et al. Bronchial thermoplasty for moderate or severe persistent asthma in adults. *Cochrane Database Syst Rev.* 2014; Issue 3.
8. Chung KF, Wenzel SE, Brozek JL, Bush A, Castro M, Sterk PJ, et al. International ERS/ATS guidelines on definition, evaluation and treatment of severe asthma. *Eur Respir J.* 2014;43(2):343-73.
9. IPG419 Bronchial thermoplasty for severe asthma [Internet]. NICE; [published January 2012, date accessed August 2018]. Available from https://www.nice.org.uk/guidance/ipg419
10. Heaney LG, Brightling CE, Menzies-Gow A, Stevenson M and Niven RM. Refractory asthma in the UK: cross-sectional findings from a UK multicentre registry. *Thorax* 2010;65:787-794.
11. Burn J, Sims AJ, Keltie K, Patrick H, Welham S, Heaney LG, Niven RM. Procedural and short-term safety of bronchial thermoplasty in clinical practice: evidence from a national registry and Hospital Episode Statistics. *J Asthma* 2017;54(8):872-879.
12. Doeing DC, Mahajan AK, White SR, Naureckas ET, Krishnan JA, Hogarth DK. Safety and feasibility of bronchial thermoplasty in asthma patients with very severe fixed airflow obstruction: a case series. *J Asthma*. 2013;50(2):215-8.
13. Fernández-Bussy S, Labarca G, Caviedes I, Folch E, Majid A. Bronchial thermoplasty for severe asthma: Initial experience in Chile. *Arch Bronconeumol*. 2016;52:52.
14. Bicknell S, Chaudhuri R, Lee N, Shepherd M, Spears M, Pitman N, et al. Effectiveness of bronchial thermoplasty in severe asthma in 'real life' patients compared with those recruited to clinical trials in the same centre. *Ther Adv Respir Dis.* 2015; 9(6):267-71.
15. Langton, David, Sha, Joy, Ing, Alvin, Fielding, David and Wood, Erica (2017). Bronchial thermoplasty in severe asthma in Australia. *Internal Medicine Journal* 19: 19.
16. Watchorn, D. C., Sahadevan, A., Egan, J. J. and Lane, S. J. (2016). The Efficacy of Bronchial Thermoplasty for Severe Persistent Asthma: The First National Experience. *Irish Medical Journal* 109(5): 406

For peer review only

17. Arrigo, Rita, Failla, Giuseppe, Scichilone, Nicola, La Sala, Alba, Galeone, Carla, Battaglia, Salvatore, Benfante, Alida and Facciolongo, Nicola (2016). How Effective and Safe Is Bronchial Thermoplasty in "Real Life" Asthmatics Compared to Those Enrolled in Randomized Clinical Trials? *BioMed Research International* 2016: 9132198

18. Chupp G, Laviolette M, Cohn L, McEvoy C, Bansal S, Shifren A, et al. Long-term outcomes of bronchial thermoplasty in subjects with severe asthma: a comparison of 3-year follow-up results from two prospective multicentre studies. *Eur Respir J.* 2017; 50(2).

19. Thomson NC and Chanez P. How effective is bronchial thermoplasty for severe asthma in clinical practice? *Eur Respir J.* 2017; 50: 1701140.

20. Barkun JS, Aronson JK, Feldman LS, Maddern GJ, Strasberg SM, Altman DG, et al. Evaluation and stages of surgical innovations. *Lancet* 2009; 374:1089-1096.

21. Commissioning through Evaluation [Internet]. NHS England; [date accessed August 2018]. Available from https://www.england.nhs.uk/commissioning/spec-services/npc-crg/comm-eval/

22. Juniper E, Guhatt G, Epstein R, Ferrie PJ, Jaeschke R, Hiller TK. Evaluation of impairment of health related quality of life in asthma: development of a questionnaire for use in clinical trials. *Thorax* 1992;47:76–83.

23. R Core Team. R: A language and environment for statistical computing [Internet]. R Foundation for Statistical Computing, Vienna, Austria; [date accessed August 2018]. Available from http://www.R-project.org/

24. Rubin DB. Multiple imputation for nonresponse in surveys. New York: John Wiley, 1987.

25. Schafer JL. Multiple imputation: a primer. *Stat Methods Med Res*. 1999 Mar;8(1):3-15.

26. White IR, Royston P, Wood AM. Multiple imputation using chained equations: Issues and guidance for practice. *Stat Med*. 2011;30(4):377–99

27. Graham, J.W., Olchowski, A.E. & Gilreath, T.D. *Prev Sci* (2007) 8: 206.

28. Pinheiro JC and Bates DM. Mixed effects models in S and S-PLUS, Springer-Verlag, New York, 2000.

For peer review only

29. Bel EH. Bronchial thermoplasty: has the promise been met? *Am J Respir Crit Care Med*. 2010;181(2):101-2.

30. Bel EH, Zwinderman AH. Outcome Reporting in Asthma Research. *Am J Respir Crit Care Med*. 2011;183(1): 132-3.

31. Iyer VN, Lim KG. Bronchial thermoplasty: reappraising the evidence (or lack thereof). *Chest*. 2014;146(1):17-21.

For peer review only

## FIGURE CAPTIONS

**Fig 1: Efficacy study participants**

**Fig 2: AQLQ series for patients with AQLQ data at BT baseline and at least one follow-up. Horizontal dashed lines show +/- 0.5 (clinically significant) change in AQLQ from BT baseline. Responders (defined as having >= 0.5 increase in AQLQ at 12 month follow-up compared with BT baseline) are shown in blue. Non-responders are shown in black.**

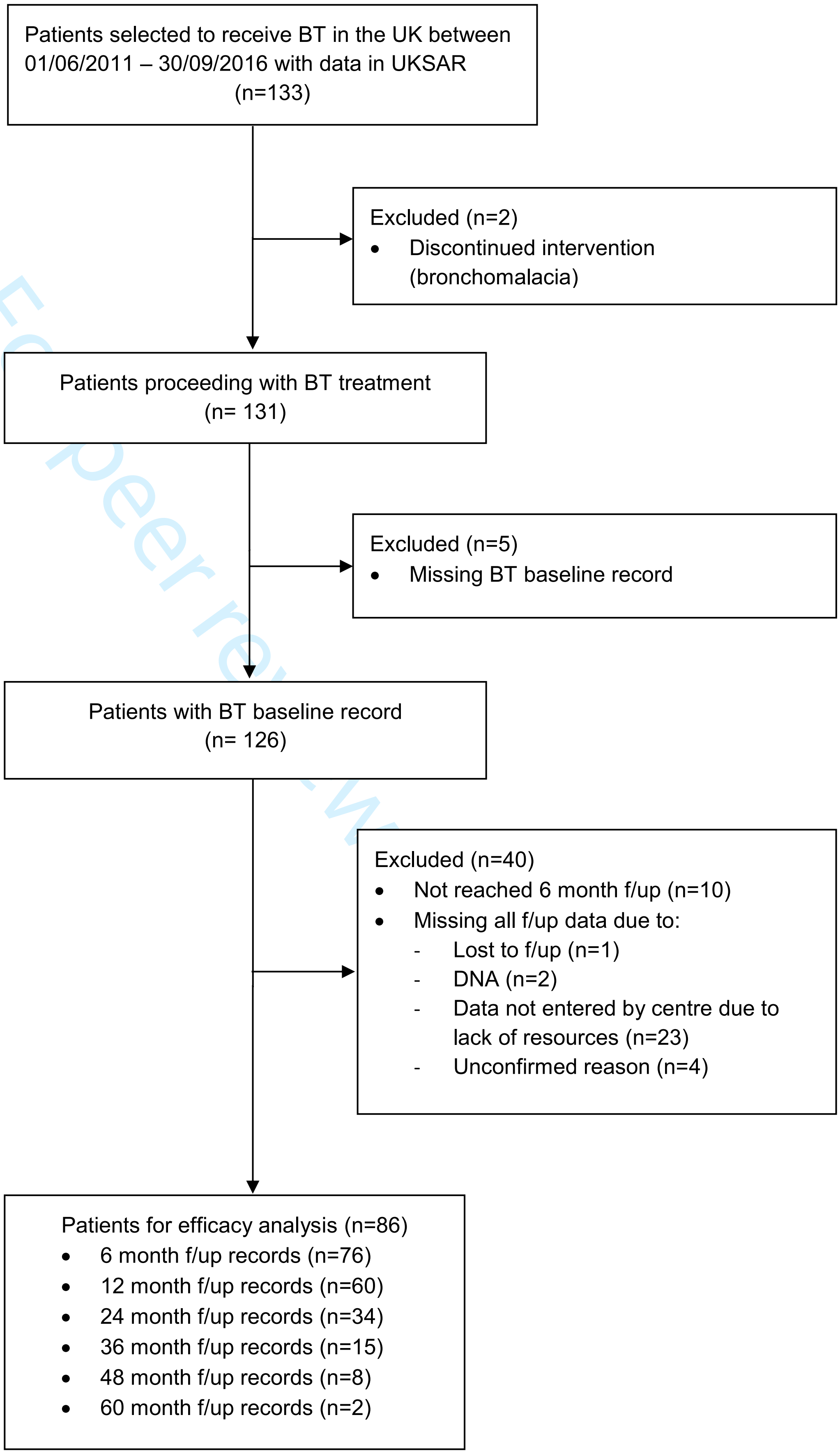


For peer review only - http://bmjopen.bmj.com/site/about/guidelines.xhtml

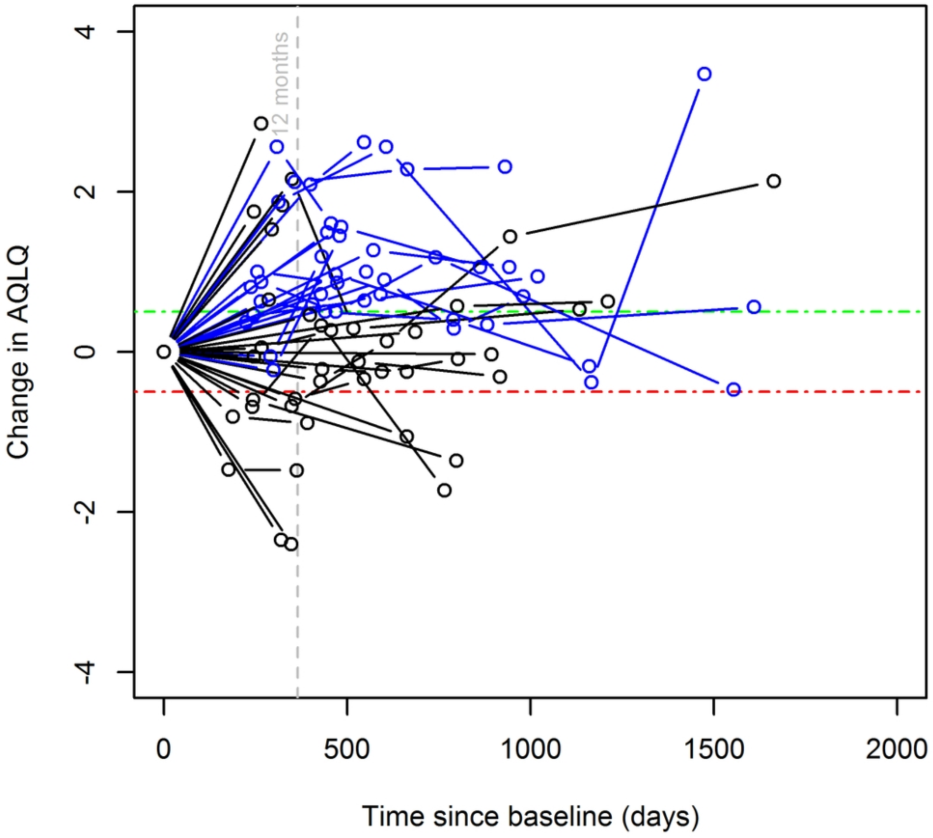


Fig 2: AQLQ series for patients with AQLQ data at BT baseline and at least one follow-up. Horizontal dashed lines show +/- 0.5 (clinically significant) change in AQLQ from BT baseline. Responders (defined as having >= 0.5 increase in AQLQ at 12 month follow-up compared with BT baseline) are shown in blue. Non-responders are shown in black.

99x99mm (300 x 300 DPI)

## Supplementary Material 1

**Efficacy and safety of bronchial thermoplasty in clinical practice: a prospective, longitudinal, cohort study using evidence from the UK Severe Asthma Registry**

**Table S1:** Data fields added to the UK Severe Asthma Registry (SAR) to collect safety and efficacy outcomes for bronchial thermoplasty (BT)

| |
|---|
| Date/time of bronchial thermoplasty |
| Procedure Number |
| Inspection of previously treated sites satisfactory (N/Y) |
| Details of previously treated airways |
| Bronchial thermoplasty procedure performed (N/Y) |
| Bronchoscopist |
| Catheter Operator |
| Technician/Nurse |
| Make/model of BT device |
| Serial number of catheter |
| Any hospital admissions since last treatment (including visits to asthma clinic) (N/Y)<br>- (If Y): Details of asthma/BT procedure related admissions<br>- (If Y): Details of other admissions |
| Prednisolone given for 5 days peri-procedure (N/30mg/40mg/50mg/60mg) |
| Was additional asthma medication prescribed because of this procedure (N/Y)<br>- (If Y): Details of other pre-treatment asthma medications |
| Site treated (RLL/LLL/RUL+LUL) |
| Number of activations |
| Missed segments: RLL (None, Posterior basal segment, Lateral basal segment, Anterior basal segment, Medial basal segment, Right lower lobe bronchus, Apical lower lobe segment) |
| Missed segments: LLL (None, Posterior basal segment, Lateral basal segment, Anterior basal segment, Left lower lobe bronchus, Apical lower lobe segment) |
| Missed segments: RUL (None, Posterior segment, Apical segment, Anterior segment, Right upper lobe bronchus) |
| Missed segments: LUL (None, Apicoposterior segment, Anterior segment, Superior division bronchus, Lingula segment, Left upper lobe bronchus) |
| Number of missed segments |
| Lowest sats peri procedure (%) |
| Duration of procedure (induction to scope out) (mins) |
| Patient discharged post procedure (N/Y)<br>- (If N): Duration of admission (<4hours/4-12hours/12-24hours/>24hours)<br>- (If >24 hours): Number of days |
| Unanticipated procedural morbidity |

For peer review only

**Table S2:** Missing data for fields used in multiple imputation analysis

| Field | BTBL missing data (n=60) | FU12 missing data (n=60) |
| --- | --- | --- |
| Hospital | 0 (0%) | 0 (0%) |
| Gender | 0 (0%) | 0 (0%) |
| Age | 0 (0%) | 0 (0%) |
| BMI | 2 (3.3%) | 15 (25%) |
| Smoking status | 4 (6.7%) | 9 (15%) |
| Eosinophil count (blood) | 17 (28.3%) | 37 (61.7%) |
| FEV1 % predicted | 3 (5%) | 8 (13.3%) |
| AQLQ score | 20 (33.3%) | 23 (38.3%) |
| EQ-5D score | 31 (51.7%) | 31 (51.7%) |
| ACQ score | 23 (38.3%) | 20 (33.3%) |
| HADS score (Anxiety) | 27 (45%) | 32 (53.3%) |
| HADS score (Depression) | 27 (45%) | 32 (53.3%) |
| Rescue steroid courses | 7 (11.7%) | 5 (8.3%) |
| Unscheduled healthcare visits[†] | 9 (15%) | 8 (13.3%) |
| Hospital admissions | 7 (11.7%) | 5 (8.3%) |

[†] Includes visits to A&E, GP and asthma clinic

Original sources describing the application of the multiple imputation technique suggested that 5 to 10 imputations were sufficient [24,25]. However, a greater number has since been recommended to avoid producing a large Monte Carlo error. White et al [26] suggested a number at least as large as the percentage of missing data and Graham, Olchowski and Gilreath [27] recommend up to 100 imputations for 70% missing data. We therefore studied the levels of missing data and set the number of imputations used in the model to 100.

Default methods of imputation were used for each variable: predictive mean matching for numeric data, logistic regression for binary data, polytomous regression for unordered categorical data and proportional odds for ordered categorical data.

For peer review only

**Table S3:** Number of records in UKSAR at 31/10/2016

| Time point | Description | Patient records in UKSAR | Patients who reached time point at 31/10/2016 |
|---|---|---|---|
| BL | Baseline (pre BT) | 126 | - |
| BT1 | First BT procedure | 128 | - |
| BT2 | Second BT procedure | 125 | - |
| BT3 | Third BT procedure | 117 | - |
| FU6 | Follow-up visit (6 months) | 76 | 86 |
| FU12 | Follow-up visit (12 months) | 60 | 82 |
| FU24 | Follow-up visit (24 months) | 34 | 64 |
| FU36 | Follow-up visit (36 months) | 15 | 38 |
| FU48 | Follow-up visit (48 months) | 8 | 22 |
| FU60 | Follow-up visit (60 months) | 2 | 5 |

For peer review only

**Table S4:** Comparison of baseline characteristics of BT patients (efficacy study). Data shown as mean (sd) or median [min,LQ,UQ,max]

| Characteristic | All patients with BTBL (n=126) | | Patients included in efficacy study (n=86) | Patients excluded from efficacy study (n=40) | P value |
|---|---|---|---|---|---|
| **Age at first BT (years)** | 43.7 (12.2) n=125 | | 41.9 (11.7) n=86 | 47.5 (12.54) n=39 | 0.020 |
| **Female (%)** | 69.8 n=126 | | 68.6 (n=86) | 72.5 n=40 | 0.657 |
| **BMI (kg/m$^2$)** | 31.8 (7.5) n=119 | | 31.2 (7.4) n=81 | 32.9 (7.8) n=38 | 0.256 |
| **Non-smoker/ Ex-smoker (%)** | 98.3 n=117 | | 97.5 n=79 | 100 n=38 | 1.0 |
| **$FEV_1$ % predicted** | 71.0 (21.8) n=118 | | 69.7 (21.7) n=82 | 74.1 (22.0) n=36 | 0.316 |
| **Eosinophil count (blood)** | 0.28 (0.34) n=89 | | 0.30 (0.37) (n=63) | 0.21 (0.21) (n=26) | 0.122 |
| **AQLQ score** | 3.66 (1.35) n= 81 | | 3.64 (1.26) n= 59 | 3.71 (1.60) n=22 | 0.848 |
| **EQ-5D score** | 0.57 (0.36) n=54 | | 0.53 (0.38) n=42 | 0.72 (0.23) n=12 | 0.049 |
| **ACQ score** | 3.19 (1.29) n=67 | | 3.28 (1.36) n=49 | 2.93 (1.06) n=18 | 0.278 |
| **HADS score (Anxiety)** | 8.13 (5.46) n=63 | | 8.52 (5.54) n=48 | 6.87 (5.19) n=15 | 0.30 |
| **HADS score (Depression)** | 6.35 (5.19) n=63 | | 6.46 (5.25) n=48 | 6.0 (5.15) n=15 | 0.767 |
| **Rescue steroid courses (previous year)** | 4 [0,2,6,15] n=100 | | 4 [0,2,5.5,15] n=75 | 5 [0,3,8,12] n=25 | 0.203 |
| **Unscheduled healthcare† (previous year)** | 5 [0,2,6,20] n=100 | | 5 [0,2,6,20] n=71 | 5 [0,3,6,15] n=29 | 0.472 |
| **Hospital admissions (previous year)** | 1 [0,0,3,11] n=103 | | 2 [0,0,3,11] n=76 | 1 [0,0,3,11] n=27 | 0.490 |

† Includes visits to A&E, GP and asthma clinic

For peer review only

**Table S5:** Available efficacy data and summary statistics at baseline and follow-up visits. Data shown as mean (sd) or median [min,LQ,UQ,max]

| | BTBL (n=86) | FU6 (n=76) | FU12 (n=60) | FU24 (n=34) | FU36 (n=15) | FU48 (n=8) | FU60 (n=2) |
|---|---|---|---|---|---|---|---|
| **AQLQ score** | 3.64 (1.26)<br>n=59 | 4.16 (1.47)<br>n=41 | 4.24 (1.45)<br>n=37 | 4.40 (1.62)<br>n=19 | 4.56 (1.57)<br>n=5 | 4.92 (2.11)<br>n=4 | -<br>n=0 |
| **EQ-5D score** | 0.53 (0.38)<br>n=42 | 0.63 (0.33)<br>n=30 | 0.62 (0.38)<br>n=29 | 0.65 (0.35)<br>n=18 | 0.79 (0.07)<br>n=4 | -<br>n=0 | -<br>n=0 |
| **ACQ score** | 3.28 (1.36)<br>n=49 | 2.72 (1.40)<br>n=47 | 2.75 (1.34)<br>n=40 | 3.06 (1.27)<br>n=21 | 2.85 (1.37)<br>n=6 | 3.1 (2.25)<br>n=3 | 5.5<br>n=1 |
| **HADS score (Anxiety)** | 8.52 (5.54)<br>n=48 | 7.73 (5.0)<br>n=33 | 6.46 (5.20)<br>n=28 | 5.28 (5.65)<br>n=18 | 5.80 (3.70)<br>n=5 | 7.0 (7.0)<br>n=3 | 19.0<br>n=1 |
| **HADS score (Depression)** | 6.46 (5.25)<br>n=48 | 5.94 (4.87)<br>n=33 | 5.07 (4.50)<br>n=28 | 4.67 (4.85)<br>n=18 | 7.0 (3.74)<br>n=5 | 6.33 (7.09)<br>n=3 | 11.0<br>n=1 |
| **FEV1 % predicted** | 69.65 (21.71)<br>n=82 | 71.47 (21.62)<br>n=59 | 74.90 (21.34)<br>n=52 | 72.71 (21.08)<br>n=31 | 76.50 (18.78)<br>n=12 | 77.86 (25.39)<br>n=7 | 81.5 (13.44)<br>n=2 |
| | | | | | | | |
| **Days last BT procedure to follow-up** | - | 190<br>[113,172.8,210.3,285]<br>n=76 | 381.5<br>[266,363.8,408,531]<br>n=60 | 738.5<br>[571,720.3,775.8,924]<br>n=34 | 1118<br>[1059,1079.5,1160.5,1228]<br>n=15 | 1508.5<br>[1367,1479.8,1524.8,1545]<br>n=8 | 1821<br>[1787,1804,1838,1855]<br>n=2 |
| **Rescue steroid courses (annualised)** | 4<br>[0,2,5.5,15]<br>n=75 | 2.0<br>[0,0,3.6,11.7]<br>n=67 | 3.1<br>[0,1.4,5.8,18.8]<br>n=55 | 1.9<br>[0,0.8,4.9,12.1]<br>n=29 | 3.1<br>[0,1.8,4.5,7.5]<br>n=14 | 2.9<br>[0,1.4,7.1,8.7]<br>n=8 | 5.8<br>[2.2,4.0,7.5,9.3]<br>n=2 |
| **Unscheduled healthcare† (annualised)** | 5<br>[0,2,6,20]<br>n=71 | 2.0<br>[0,0,4.9,12.1]<br>n=61 | 3.2<br>[0,1.1,5.6,11.5]<br>n=52 | 1.3<br>[0,0,2.9,11.8]<br>n=29 | 3.0<br>[0,2.0,5.1,10.6]<br>n=14 | 4.1<br>[0,0,5.3,6.1]<br>n=8 | 6.3<br>[3.3,4.8,7.8,9.3]<br>n=2 |
| **Hospital admissions (annualised)** | 2<br>[0,0,3,11]<br>n=76 | 0<br>[0,0,0,7.2]<br>n=68 | 0<br>[0,0,1.9,11.2]<br>n=55 | 0<br>[0,0,0.6,3.9]<br>n=29 | 0<br>[0,0,1.0,4.2]<br>n=14 | 0<br>[0,0,0,4.8]<br>n=8 | 0.7<br>[0,0.3,1.0,1.3]<br>n=2 |

† Includes visits to A&E, GP and asthma clinic

For peer review only

**Fig S1:** Efficacy data (continuous variables) at baseline and follow-ups, showing mean and 95% confidence intervals of the mean

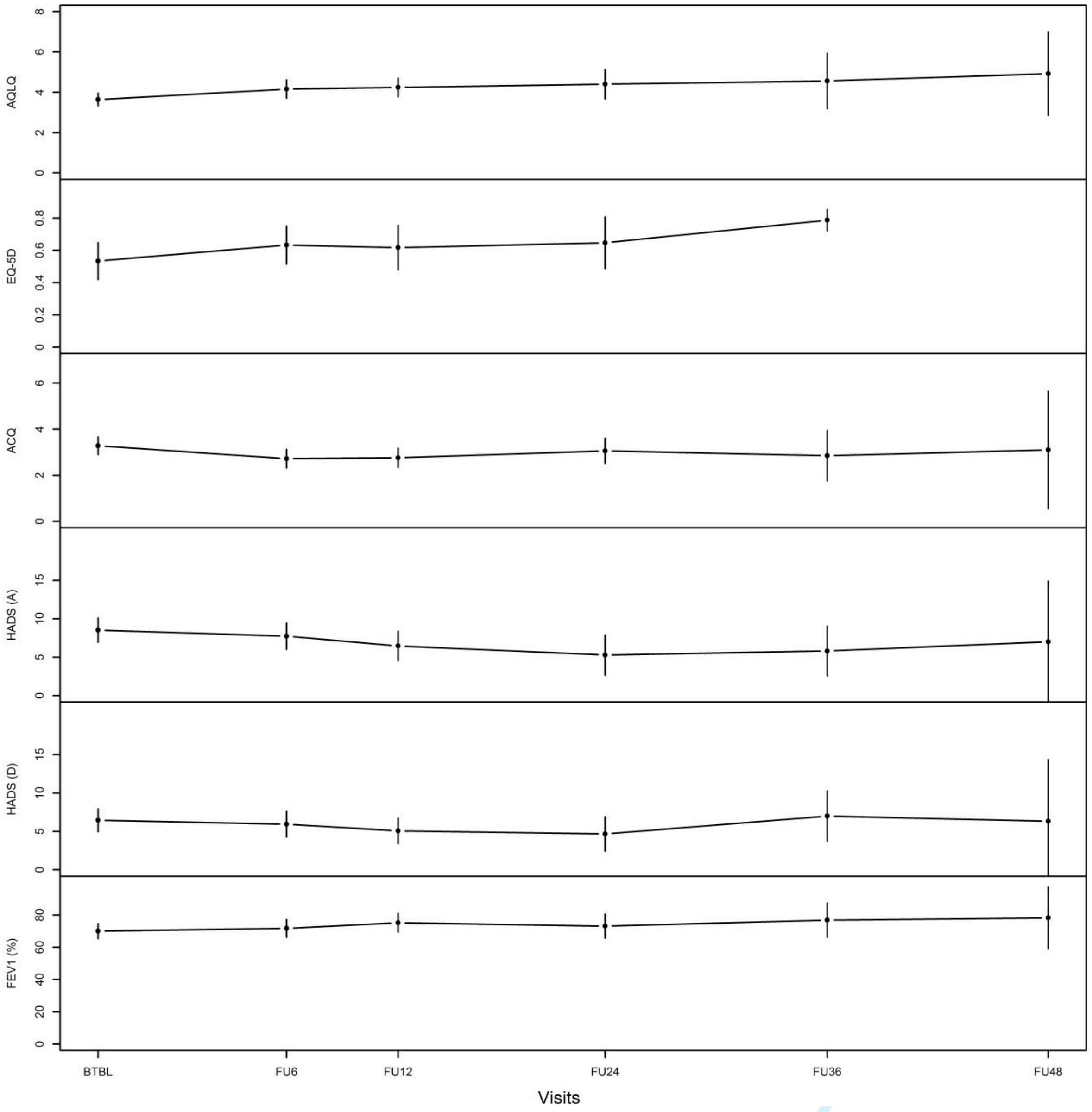

```
Multiple Imputation using MICE package: BTBL -> FU12 AQLQ analysis:
==================================================================
**Number of imputations= 100 , Number of iterations=5 (default), imputation method=default, seed=500**

Columns included in imputation:
-------------------------------
Hospital, Gender, BT1_Age, BL_BMI, BL_SmokingStatus, BL_EosinophilCount_blood, BL_FEV1_Predicted, BL_AQLQ,
BL_Eq_5d, BL_ACQ, BL_HadsA, BL_HadsD, BL_RescueSteroidCourses, BL_UnscheduledHealthcareVisits,
BL_TotalNumberOfHospitalAdmissions, FU12_Age, FU12_BMI, FU12_SmokingStatus, FU12_EosinophilCount_blood,
FU12_FEV1_Predicted, FU12_AQLQ, FU12_Eq_5d, FU12_ACQ, FU12_HadsA, FU12_HadsD, FU12_Annualised_RescueSteroids,
FU12_Annualised_UnscheduledHC, FU12_Annualised_HospAdmissions

1. Paired t-tests
=================
60 patients have a 12 month follow-up record and are included in the non-imputed/imputed analysis below

a) Paired t-test performed using non-imputed data:
==================================================
BTBL n= 40 (mean AQLQ = 3.557 )
FU12 n= 37 (mean AQLQ = 4.243 )
Paired n= 28 (mean AQLQ diff= 0.7507143 , p= 0.0002593749 )


b) Paired t-test performed using MICE:
======================================
    AQLQ_BTBL_mean AQLQ_FU12_mean AQLQ_change_mean AQLQ_change_sd      p_value
1            3.679          4.201            0.522          1.021 2.046333e-04
2            3.626          4.234            0.608          1.398 1.320145e-03
3            3.696          4.367            0.671          0.957 1.092763e-06
4            3.418          4.089            0.671          1.001 2.744490e-06
5            3.551          4.224            0.674          1.027 4.068589e-06
6            3.508          4.239            0.731          1.014 6.228038e-07
7            3.584          4.153            0.570          1.037 7.558415e-05
8            3.526          4.143            0.617          1.160 1.194300e-04
9            3.578          4.289            0.710          1.144 1.082341e-05
10           3.529          4.255            0.726          1.027 9.422217e-07
11           3.505          4.272            0.767          0.944 4.193465e-08
12           3.623          4.038            0.415          1.353 2.091213e-02
13           3.568          4.153            0.585          1.024 4.194729e-05
14           3.525          4.075            0.550          1.141 4.224508e-04
15           3.671          4.241            0.570          1.360 1.924222e-03
16           3.559          4.150            0.592          1.114 1.225935e-04
17           3.567          4.239            0.673          1.064 7.975311e-06
18           3.555          4.410            0.855          1.041 3.212725e-08
19           3.556          4.418            0.862          1.044 2.819920e-08
20           3.645          4.318            0.673          1.116 1.780862e-05
21           3.591          4.200            0.609          0.978 1.028893e-05
22           3.677          4.202            0.525          1.028 2.058468e-04
23           3.403          4.220            0.817          1.008 4.452123e-08
24           3.499          4.087            0.588          1.096 1.061262e-04
25           3.513          4.242            0.729          1.130 5.514240e-06
```

| | | | | | |
|---|---|---|---|---|---|
| 26 | 3.368 | 4.361 | 0.992 | 1.228 | 4.745462e-08 |
| 27 | 3.578 | 4.219 | 0.641 | 0.984 | 4.659109e-06 |
| 28 | 3.554 | 4.156 | 0.602 | 1.174 | 1.955385e-04 |
| 29 | 3.544 | 4.327 | 0.783 | 1.164 | 2.523290e-06 |
| 30 | 3.549 | 4.268 | 0.718 | 1.135 | 7.738990e-06 |
| 31 | 3.596 | 4.145 | 0.549 | 1.093 | 2.564226e-04 |
| 32 | 3.579 | 4.296 | 0.717 | 1.104 | 4.923972e-06 |
| 33 | 3.572 | 4.193 | 0.621 | 0.952 | 4.548948e-06 |
| 34 | 3.583 | 4.336 | 0.753 | 1.037 | 5.419597e-07 |
| 35 | 3.499 | 4.308 | 0.809 | 1.324 | 1.418903e-05 |
| 36 | 3.883 | 3.937 | 0.054 | 1.660 | 8.031300e-01 |
| 37 | 3.636 | 4.356 | 0.720 | 1.123 | 6.188136e-06 |
| 38 | 3.627 | 4.189 | 0.562 | 1.243 | 8.864559e-04 |
| 39 | 3.523 | 4.284 | 0.761 | 1.117 | 1.974242e-06 |
| 40 | 3.711 | 4.327 | 0.616 | 1.100 | 5.726456e-05 |
| 41 | 3.442 | 4.178 | 0.736 | 1.162 | 7.744180e-06 |
| 42 | 3.571 | 4.053 | 0.482 | 1.093 | 1.168465e-03 |
| 43 | 3.693 | 4.265 | 0.572 | 1.033 | 6.652695e-05 |
| 44 | 3.614 | 4.287 | 0.673 | 1.135 | 2.321598e-05 |
| 45 | 3.431 | 4.153 | 0.722 | 1.064 | 2.157225e-06 |
| 46 | 3.764 | 4.263 | 0.498 | 1.098 | 8.500630e-04 |
| 47 | 3.623 | 4.162 | 0.539 | 1.094 | 3.311606e-04 |
| 48 | 3.469 | 4.204 | 0.734 | 1.047 | 1.099687e-06 |
| 49 | 3.601 | 4.002 | 0.401 | 1.336 | 2.366637e-02 |
| 50 | 3.652 | 4.245 | 0.593 | 1.185 | 2.698488e-04 |
| 51 | 3.608 | 4.204 | 0.596 | 1.183 | 2.494405e-04 |
| 52 | 3.846 | 4.321 | 0.476 | 1.323 | 7.174397e-03 |
| 53 | 3.572 | 4.117 | 0.545 | 1.110 | 3.446290e-04 |
| 54 | 3.533 | 4.511 | 0.978 | 0.981 | 1.574084e-10 |
| 55 | 3.637 | 4.181 | 0.544 | 1.363 | 3.052196e-03 |
| 56 | 3.470 | 4.147 | 0.677 | 1.056 | 6.109291e-06 |
| 57 | 3.631 | 4.206 | 0.575 | 1.088 | 1.300066e-04 |
| 58 | 3.626 | 4.214 | 0.587 | 1.163 | 2.387147e-04 |
| 59 | 3.592 | 4.359 | 0.766 | 1.255 | 1.441879e-05 |
| 60 | 3.459 | 4.306 | 0.847 | 1.086 | 1.111670e-07 |
| 61 | 3.586 | 4.636 | 1.050 | 1.384 | 2.070639e-07 |
| 62 | 3.647 | 4.437 | 0.790 | 1.176 | 2.597081e-06 |
| 63 | 3.634 | 4.139 | 0.505 | 1.157 | 1.291805e-03 |
| 64 | 3.444 | 4.135 | 0.691 | 1.349 | 2.005913e-04 |
| 65 | 3.533 | 4.310 | 0.777 | 1.045 | 3.184540e-07 |
| 66 | 3.564 | 4.285 | 0.721 | 1.042 | 1.425469e-06 |
| 67 | 3.667 | 4.283 | 0.616 | 0.984 | 9.377156e-06 |
| 68 | 3.415 | 4.263 | 0.848 | 1.114 | 1.880069e-07 |
| 69 | 3.535 | 4.055 | 0.520 | 1.070 | 3.867826e-04 |
| 70 | 3.567 | 4.230 | 0.662 | 1.087 | 1.508066e-05 |
| 71 | 3.563 | 4.238 | 0.675 | 1.060 | 7.027794e-06 |
| 72 | 3.629 | 4.324 | 0.694 | 1.075 | 5.403097e-06 |
| 73 | 3.654 | 4.206 | 0.553 | 1.156 | 4.728816e-04 |
| 74 | 3.529 | 4.322 | 0.793 | 1.124 | 9.885601e-07 |
| 75 | 3.559 | 4.145 | 0.586 | 1.068 | 7.792340e-05 |
| 76 | 3.597 | 4.182 | 0.586 | 1.250 | 5.977232e-04 |

```
77          3.627         4.365             0.738         1.083 1.969082e-06
78          3.518         4.126             0.608         0.963 8.083937e-06
79          3.521         4.233             0.712         1.208 2.599152e-05
80          3.610         4.279             0.670         0.923 5.504651e-07
81          3.534         4.138             0.604         1.080 5.789996e-05
82          3.599         4.161             0.562         1.150 3.621040e-04
83          3.619         4.208             0.589         1.457 2.695669e-03
84          3.553         4.270             0.717         1.248 3.889592e-05
85          3.647         4.381             0.734         1.346 8.421207e-05
86          3.525         4.242             0.718         0.994 6.041832e-07
87          3.590         4.230             0.640         0.943 2.132386e-06
88          3.505         4.099             0.594         1.061 5.770850e-05
89          3.469         4.248             0.779         1.038 2.615707e-07
90          3.619         4.077             0.458         1.260 6.644815e-03
91          3.571         4.133             0.561         1.146 3.526227e-04
92          3.704         4.201             0.497         0.922 9.906683e-05
93          3.722         4.488             0.766         1.278 1.969742e-05
94          3.576         4.155             0.579         1.188 3.696589e-04
95          3.577         4.250             0.674         1.322 2.128204e-04
96          3.489         4.272             0.783         1.122 1.218046e-06
97          3.590         4.259             0.670         1.001 2.797319e-06
98          3.529         4.385             0.856         0.906 7.912775e-10
99          3.664         4.403             0.738         1.124 3.974174e-06
100         3.679         4.304             0.626         1.048 2.117619e-05


Pooled results:
----------------
 AQLQ_BTBL_mean AQLQ_BTBL_sd AQLQ_FU12_mean AQLQ_FU12_sd AQLQ_change_mean AQLQ_change_sd    min_P_val max_P_val count_p_nonsig
          3.579        0.085          4.236         0.11            0.657          0.134 1.574084e-10   0.80313              1
---------------------------------------------------------------------------------------------------------------------------------

2. Multiple linear regression (MLR)
===================================
60 patients have a 12 month follow-up record and are included in the non-imputed/imputed analysis below

a) MLR performed using non-imputed data (using median values for centering):
============================================================================
BTBL n= 40 (mean AQLQ = 3.557 )
FU12 n= 37 (mean AQLQ = 4.243 )
paired n = 28
MLR modelled the outcome as the change in AQLQ from BT baseline to 12 month follow-up with covariates:
age at first BT procedure, BMI at BT baseline, FEV1 (% predicted) at BT baseline and eosinophil count (blood) at BT baseline

Call:
lm(formula = AQLQdiff ~ Adj_BT1_CALC_Age + Adj_BL_CALC_Bmi + 
    Adj_BL_CALC_FEV1_Predicted + Adj_BL_EosinophilCount_blood, 
    data = AQLQ_DIFF)

Residuals:
     Min       1Q   Median       3Q      Max 
```

```
-1.31511 -0.31958 -0.02134  0.42481  1.09010

Coefficients:
                             Estimate Std. Error t value Pr(>|t|)    
(Intercept)                  0.787139   0.161073   4.887 0.000165 ***
Adj_BT1_CALC_Age            -0.063336   0.012883  -4.916 0.000155 ***
Adj_BL_CALC_Bmi             -0.049952   0.025250  -1.978 0.065374 .  
Adj_BL_CALC_FEV1_Predicted   0.013192   0.007707   1.712 0.106266    
Adj_BL_EosinophilCount_blood -0.038687   0.526264  -0.074 0.942309    
---
Signif. codes:  0 ‘***’ 0.001 ‘**’ 0.01 ‘*’ 0.05 ‘.’ 0.1 ‘ ’ 1

Residual standard error: 0.6251 on 16 degrees of freedom
  (7 observations deleted due to missingness)
Multiple R-squared:  0.6721,    Adjusted R-squared:  0.5901 
F-statistic: 8.199 on 4 and 16 DF,  p-value: 0.0008522


b) MLR using MICE:
==================
                                est          se           t       df   Pr(>|t|)       lo 95       hi 95 nmis       fmi    lambda
(Intercept)               2.255356211 1.310516486  1.72096745 27.49264 0.09649391 -0.43134875 4.942061173   NA 0.4872430 0.4512508
BT1_CALC_Age             -0.028672937 0.015566560 -1.84195714 28.99557 0.07573107 -0.06051034 0.003164465    0 0.4597051 0.4236801
BL_CALC_BMI              -0.030466048 0.024170045 -1.26048783 29.27763 0.21744708 -0.07987899 0.018946892    2 0.4545732 0.4185448
BL_CALC_FEV1_Predicted    0.008620545 0.009267073  0.93023383 27.04739 0.36047888 -0.01039236 0.027633448    3 0.4954656 0.4594883
BL_EosinophilCount_blood -0.076979354 0.788169309 -0.09766855 26.05245 0.92294303 -1.69692585 1.542967139   17 0.5139486 0.4780146
```

For peer review only - http://bmjopen.bmj.com/site/about/guidelines.xhtml

STROBE Statement—Checklist of items that should be included in reports of ***cohort studies***

| | Item No | Recommendation | Document reference |
|---|---|---|---|
| **Title and abstract** | 1 | (*a*) Indicate the study's design with a commonly used term in the title or the abstract | Title and abstract |
| | | (*b*) Provide in the abstract an informative and balanced summary of what was done and what was found | Abstract |
| **Introduction** | | | |
| Background/rationale | 2 | Explain the scientific background and rationale for the investigation being reported | Pages 4-5 |
| Objectives | 3 | State specific objectives, including any prespecified hypotheses | Page 5 |
| **Methods** | | | |
| Study design | 4 | Present key elements of study design early in the paper | Page 6 |
| Setting | 5 | Describe the setting, locations, and relevant dates, including periods of recruitment, exposure, follow-up, and data collection | Page 6 |
| Participants | 6 | (*a*) Give the eligibility criteria, and the sources and methods of selection of participants. Describe methods of follow-up | Page 6 |
| | | (*b*) For matched studies, give matching criteria and number of exposed and unexposed | N/A |
| Variables | 7 | Clearly define all outcomes, exposures, predictors, potential confounders, and effect modifiers. Give diagnostic criteria, if applicable | Pages 7- 8 |
| Data sources/ measurement | 8* | For each variable of interest, give sources of data and details of methods of assessment (measurement). Describe comparability of assessment methods if there is more than one group | Pages 6 -7 |
| Bias | 9 | Describe any efforts to address potential sources of bias | Pages 7-10 |
| Study size | 10 | Explain how the study size was arrived at | Page 6 |
| Quantitative variables | 11 | Explain how quantitative variables were handled in the analyses. If applicable, describe which groupings were chosen and why | Page 8 |
| Statistical methods | 12 | (*a*) Describe all statistical methods, including those used to control for confounding | Pages 8-10 |
| | | (*b*) Describe any methods used to examine subgroups and interactions | Pages 9-10, 19 |
| | | (*c*) Explain how missing data were addressed | Pages 8-9 |
| | | (*d*) If applicable, explain how loss to follow-up was addressed | Fig 1, n provided in all results tables) |
| | | (*e*) Describe any sensitivity analyses | Page 7,8,9 |
| **Results** | | | |
| Participants | 13* | (a) Report numbers of individuals at each stage of study—eg numbers potentially eligible, examined for eligibility, confirmed eligible, included in the study, completing follow-up, and analysed | Page 10 and Fig 1 |
| | | (b) Give reasons for non-participation at each stage | Fig 1 |
| | | (c) Consider use of a flow diagram | Fig 1 |


For peer review only - http://bmjopen.bmj.com/site/about/guidelines.xhtml

| | | | |
|---|---|---|---|
| Descriptive data | 14* | (a) Give characteristics of study participants (eg demographic, clinical, social) and information on exposures and potential confounders | Page 10, Table 1, Table S4 |
| | | (b) Indicate number of participants with missing data for each variable of interest | Table S3,S4 (n provided in all results tables) |
| | | (c) Summarise follow-up time (eg, average and total amount) | Page 10 |
| Outcome data | 15* | Report numbers of outcome events or summary measures over time | Pages 11,12, 16,17 Tables 2,3,4,6,S5 Fig 2 |
| Main results | 16 | (*a*) Give unadjusted estimates and, if applicable, confounder-adjusted estimates and their precision (eg, 95% confidence interval). Make clear which confounders were adjusted for and why they were included | Table 2 |
| | | (*b*) Report category boundaries when continuous variables were categorized | Table 2 |
| | | (*c*) If relevant, consider translating estimates of relative risk into absolute risk for a meaningful time period | N/A |
| Other analyses | 17 | Report other analyses done—eg analyses of subgroups and interactions, and sensitivity analyses | Pages 14,15 Table 5 |
| **Discussion** | | | |
| Key results | 18 | Summarise key results with reference to study objectives | Pages 18,19,20 |
| Limitations | 19 | Discuss limitations of the study, taking into account sources of potential bias or imprecision. Discuss both direction and magnitude of any potential bias | Pages 20,21 |
| Interpretation | 20 | Give a cautious overall interpretation of results considering objectives, limitations, multiplicity of analyses, results from similar studies, and other relevant evidence | Page 21 |
| Generalisability | 21 | Discuss the generalisability (external validity) of the study results | Page 21 |
| **Other information** | | | |
| Funding | 22 | Give the source of funding and the role of the funders for the present study and, if applicable, for the original study on which the present article is based | Page 23 |

*Give information separately for exposed and unexposed groups.

**Note:** An Explanation and Elaboration article discusses each checklist item and gives methodological background and published examples of transparent reporting. The STROBE checklist is best used in conjunction with this article (freely available on the Web sites of PLoS Medicine at http://www.plosmedicine.org/, Annals of Internal Medicine at http://www.annals.org/, and Epidemiology at http://www.epidem.com/). Information on the STROBE Initiative is available at http://www.strobe-statement.org.

For peer review only
